# Non-concussive head impacts sustained during American football correlate with changes in gut microbiome diversity and composition

**DOI:** 10.1371/journal.pone.0345651

**Published:** 2026-05-06

**Authors:** Zachary J. Pelland, Aziz Zafar, Ahmet A. Ay, Kenneth Douglas Belanger

**Affiliations:** 1 Program in Neuroscience, Colgate University, Hamilton, New York, United States of America; 2 Department of Biology, Colgate University, Hamilton, New York, United States of America; 3 Department of Mathematics, Colgate University, Hamilton, New York, United States of America; Sun Yat-Sen University, CHINA

## Abstract

Non-concussive head impacts (NHIs) are a significant health concern among at-risk groups, including athletes and military personnel. NHIs are hits to the head or head acceleration events (HAEs) that do not generate clinically detectable symptoms and are unlikely to meet diagnostic criteria for mild traumatic brain injury (mTBI). The composition of the gut microbiota influences many aspects of health and wellness and can be altered by TBIs and by brain-related diseases and disorders; however, microbiome alterations have not previously been linked to NHIs. We investigated whether NHIs in a cohort of American football players correlate with acute and long-term changes in the gut microbiome. This study monitored head impact exposure, gut microbiome composition, and a breadth of clinical and behavioral factors in a cohort of collegiate American football players across a competition season. Both short- and long-term changes in the microbiome were analyzed for correlation with head impact events and mathematical modeling was used to examine the contribution of NHIs and other clinical factors to these changes. We observe that NHI exposure correlates with changes in microbial diversity and composition three days following a head impact event. Furthermore, the athletes’ gut microbiomes change significantly across the season, with evidence from mixed-effects modeling indicating that the cumulative effects of NHIs contribute to this change. Our results provide strong evidence for a link between NHIs and changes in the diversity and composition of the gut microbiome. The outcomes of this study emphasize the importance of careful monitoring of head impacts, including those that do not generate clinical symptoms.

## Introduction

Mild traumatic brain injuries (mTBIs) are acute injuries to the brain caused by external physical forces applied to the head or secondary forces applied to other parts of the body, commonly resulting in confusion, a brief loss of consciousness, amnesia, and other transient neurological symptoms [1]. Approximately 1 in 6 mTBI patients are subject to short-term disabilities caused by symptoms prolonging for three or more months following an injury [2,3], and repeated exposure to mTBIs—common among military personnel and contact sport athletes [4]—has been linked to chronic disability and an increased risk of developing neurodegenerative and psychiatric diseases (reviewed in [5–7]).

The short-term disabilities and chronic neurodegenerative pathologies that may develop after exposure(s) to mTBIs have been linked to chronic neuro- and systemic inflammation (reviewed in [8,9]). The gut microbiome, the community of trillions of microorganisms that colonize the intestinal tract, is a key regulator of inflammation and the neuroimmune system [10,11]. Numerous neurological conditions with similar inflammatory characteristics as mTBI have been associated with abnormalities in the composition and disruption to the homeostasis of the gut microbiome, known as gut dysbiosis in humans and rodent models [12,13]. Recent findings suggest that mTBIs similarly cause gut dysbiosis (reviewed in [14]), which may play a role in the short-term disability and the development of neurodegeneration linked to repetitive mTBIs [15–17]. Identifying and treating gut dysbiosis has shown the potential to diagnose and alleviate the symptoms of other neurological and psychiatric conditions [18–20]. Thus, recognizing and characterizing alterations in the gut microbiome following mTBIs represents a potential avenue to predict, treat, and prevent severe or long-term neurological damage from mTBIs [21–23].

While mTBIs have been more substantially explored, non-concussive head impacts (NHIs) have been less so, given the challenges in monitoring these injuries. NHIs consist of cranial impacts or head acceleration events (HAEs) that likely contribute to minor brain injuries but are not recognized or clinically diagnosed as an mTBI [24,25]. Repeated exposure to NHIs is particularly common among American football players, with athletes experiencing between 100 and 1,000 head impacts across a season [24,26,27]. Similar to mTBIs, the accumulation of NHIs has been linked to acute changes in inflammatory markers, short-term changes in cognitive function, and an increased risk of neurodegeneration or early cognitive decline. Given the overlaps between mTBIs and NHIs, NHIs may also alter the gut microbiome, potentially contributing to acute and long-term harms of NHIs [28–33]. However, to our knowledge, a link between NHIs and gut microbiome composition has not yet been investigated.

In this study, we tested two hypotheses: whether individual or accumulated HAEs on a single day correlate with acute changes in the gut microbiome, and whether NHIs have a cumulative effect that leads to longer-term changes in the gut microbiome. To test these hypotheses, we monitored the gut microbiomes of National Collegiate Athletic Association (NCAA) Division I American football players across a competition season while tracking HAEs sustained during all practices and games. We found that microbial diversity in athletes changed within three days after substantial nonconcussive head impact exposure. Specifically, we observed correlations between changes in the abundance of Coriobacteriales, *Prevotella*, and *Ruminococcus* and head impact load sustained in the past 48–72 hours. We also found evidence that the athletes’ gut microbiomes changed significantly across the season and that this change may be partially due to the cumulative effects of NHIs and other clinical factors. These data provide the first evidence for a link between NHIs and acute and long-term changes in the gut microbiome.

## Materials and methods

### Study overview

**Fig 1:**
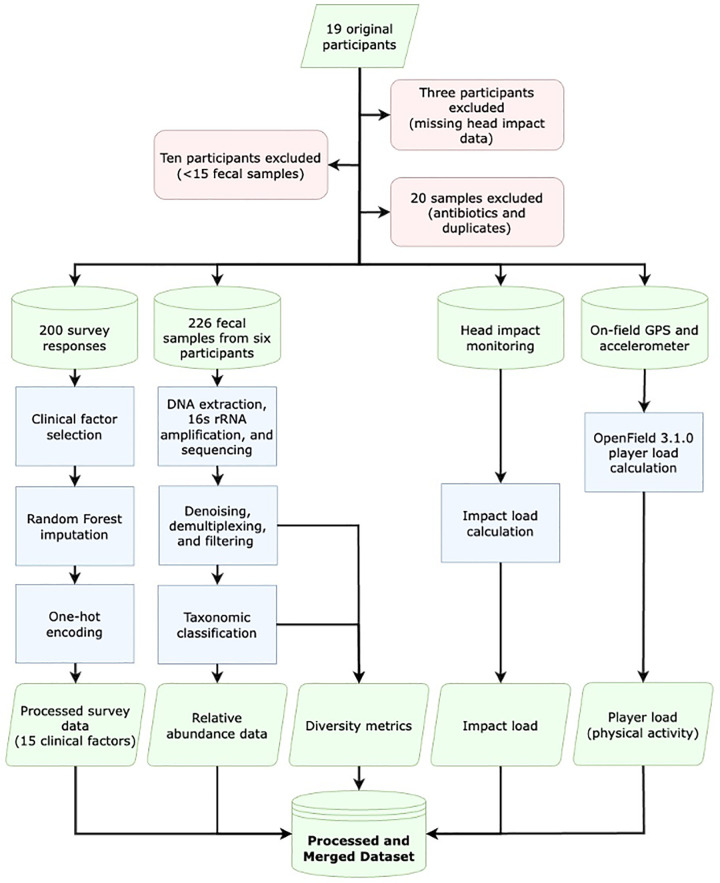
Data collection and processing flowchart. Head impact data, on-field activity data, surveys of clinically relevant factors, and fecal samples were collected from 19 original participants. Thirteen participants were excluded due to missing head impact data or an insufficient number of fecal samples (fewer than 15 samples). Twenty fecal samples from the remaining six participants were excluded due to oral antibiotic prescription or duplicate samples collected on the same day. Fifteen clinical factors from available survey data were selected, and missing survey data were imputed. After exclusions, the bacterial DNA from 226 fecal samples was extracted, sequenced, and processed into relative abundance data and diversity metrics. Head impacts were monitored, and impact load was calculated based on the severity of impacts. On-field activity was monitored to measure physical strain (player load).

Nineteen NCAA Division I American football team members had their head impacts, on-field physical activity, relevant clinical factors, and fecal microbiome monitored across a competition season, beginning during preseason training (Fig 1). After exclusions, 226 fecal samples from the six remaining participants were analyzed. Head impacts were monitored using the Riddell_®_ InSite helmet-based impact monitoring system (Riddell_®_), on-field physical activity with portable 10 Hz GPS units (Catapult S7 and G7, Catapult Sports), clinical factors were reported through digital surveys, and the fecal microbiome was characterized with 16S rRNA PCR amplification and next-generation sequencing.

### Participants

This single-site prospective cohort study recruited 19 active male NCAA Division I Football Championship Subdivision team members through a survey sent to the team (~90 players). Participants were excluded from the study if they had been prescribed oral antibiotics or had sustained a diagnosed concussion in the past 60 days. Three of the initial 19 subjects were excluded from analysis due to all or part of their head impact data not being collected, and ten more subjects were excluded due to collecting fewer than 15 fecal samples. Only the data for the six remaining participants were analyzed. Prior to sample collection, participants reported medical history, sports history, and demographic information (see S1 File for the survey). The on-field positions of the participants will not be provided to protect participant confidentiality. All six participants were male, white or Caucasian, 21 or 22 years old, and received the majority of their meals from the same source (see S2 Table for further demographics). All study procedures were approved by the Institutional Review Board of Colgate University as proposal FR-S22-13. Written consent was obtained from all participants prior to data collection.

### Head impact data

Head impact data was collected using the Riddell_®_ InSite helmet-based impact monitoring system (Riddell_®_). The Riddell_®_ InSite system includes player units and helmet liners that track and characterize impacts to the helmet based on the linear force at one of five levels (15–19 g, 20–28 g, 29–43 g, 44–63 g, and 63 + g) and location in one of five regions of the helmet (front, crown, back, left, and right). In the present study, an impact load score was calculated by granting head impacts between 15–19 g a score of 17, 20–28 g a score of 24, 29–43 g a score of 36, 44–63 g a score of 53.5, and 63 + g a score of 63. The sum of these scores for head impacts recorded during each practice and game was calculated to generate a “head impact load” for each session. Practice and game sessions were designated a specific time point to determine when head impacts were sustained in reference to fecal sample collections; the time point for each head impact load score was designated to be the midpoint of the practice or game (i.e., head impacts sustained during a practice session from 4:00 PM to 6:00 PM were designated to have occurred at 5:00 PM). Head impact location was not taken into consideration for this study.

### On-field activity monitoring

The activity profiles of four of the six participants and 14 total team members were monitored during every training and competition session with portable 10 Hz GPS units (Catapult S7 and G7, Catapult Sports). The GPS unit was worn in a vest and rested between the shoulder blades without limiting upper limb or torso movements. In addition to the GPS-derived velocity, distance, and acceleration values, the GPS units also provided inertial movement analysis (IMA) data based on integrating 100 Hz microsensor data (accelerometer, gyroscope, and magnetometer). The data was processed using dedicated software (Catapult OpenField Version 3.1.0) to generate the player load metric, which was then exported for analysis. Participants 8 and 16 did not wear designated activity monitors, and the team average player load (calculated from the 14 team members) was assigned to them for each practice and game session.

### Fecal sample collection lifestyle questionnaires

Participants were asked to complete a brief questionnaire following each fecal sample collection and comprehensive background surveys before and after the season. The daily questionnaires (S1 and S2 Files) assessed clinical factors pertaining to the experiences and behaviors of the participants during the 24 hours before each fecal sample collection. Some clinical factors were excluded from the analysis due to low or inaccurate reporting. Fifteen clinical factors were kept in the final analysis, including a stress rating modified from the PSS-10 [34], a selection of SCAT5 [35] symptoms, sleep quantity, sleep quality, illness, vomiting, caffeine use, NSAID use, supplement use, alcohol use, nicotine use, Bristol Stool Form Scale [36], orthopedic injury, and other potentially relevant factors (S1 Table). Participants occasionally did not complete the questionnaire after a sample collection. For fecal samples not accompanied by a questionnaire, the survey data was imputed using the miceRanger library in R. When the time of day of fecal sample collection was not recorded, the participant’s average time of day of collection was used.

### Fecal sample collection and storage

Participants were instructed to collect fecal samples from daily bowel movements using the Norgen Biotek Corp Stool Collection Kit (Norgen Biotek Corp) or Protocult^TM^ Collection Device (Therapak, LLC). All fecal samples were submerged in Norgen Biotek nucleic acid preservative (Norgen Biotek Corp) immediately after collection and stored at −20°C. A total of 246 fecal samples were collected from the remaining six participants throughout the study. Fifteen fecal samples from participant 9 and four samples from participant 16 were excluded from the analysis after they were prescribed oral antibiotics. Participant 9 collected two fecal samples on a single day, and the average taxonomic and diversity data was calculated from these samples, leaving 226 total fecal samples for analysis.

### DNA extraction and sequencing

DNA was extracted from the fecal samples using the DNeasy PowerSoil Kit (Qiagen) according to the manufacturer’s instructions. 16S rRNA PCR amplification and next-generation sequencing were performed at MR DNA (www.mrdnalab.com) using primers 515F-Y (5’-GTGCCAGCMGCCGCGGTAA-3’) [37] and 806R (5’-GGACTACHVGGTWTCTAAT-3’) [38] using Illumina MiSeq (Illumina Corp) 2x300 paired-end reads. Control samples were sequenced, including an internal mock (ATCC), a negative control that went through the extraction process, a negative control consisting of a stock solution, and two positive controls consisting of *Shigella spp.* (S1 Fig). We obtained 14,276,903 16S rRNA V4 region sequences across the 226 samples, yielding an average of 58,036 (SD 18,033) reads per sample. The minimum forward and backward trimmed sequence lengths used in the analysis were 220 and 180, respectively.

### Data processing – Taxonomic data

For each sample, forward and reverse reads were processed using QIIME2’s processing pipeline [39]. The DADA2 method was used for denoising, with 220 base pairs truncated for the forward read and 180 for the reverse read. The truncation length was identified through visual assessment, balancing a trade-off between information and the data size. For the obtained amplicon sequence variants (ASVs), any features that appeared in fewer than three samples or had an absolute abundance below ten were removed. ASVs were collapsed into taxonomic levels using the Silva V4 Classifier [40]. The classifier provided an abundance table for Operational Taxonomic Units (OTUs), such as specific microbial orders or species. The relative abundances of OTUs were computed for each sample and used for further analysis. Due to our limited sample size, analysis was done on the eight most abundant orders and twenty-one specific taxa (1 order, 11 families, 8 genera, and 1 species) reported to be significantly related to brain injuries in the literature [41–47] and available in the present dataset (S3 File).

### Data processing – Alpha and beta diversity

QIIME2 was used to compute Faith’s Phylogenetic Diversity using ASVs directly with an RAxML phylogenetic tree and Simpson’s Diversity using species-level abundances for each stool sample to measure alpha diversity within each sample [48]. Faith’s Phylogenetic Diversity was calculated by first generating a phylogenetic tree based on sequence similarities [49]. Beta diversity was calculated for each sample collected with respect to that individual’s baseline sample collected before the first preseason practice session (Day 1) using Bray-Curtis Dissimilarity measurement [50].

### Data analysis – Data slicing and repeated-measures analysis

To understand the association between head impact and diversity metrics over time, we identified practice and game sessions in our data in which a player sustained a head impact load at or above the 75th percentile of all head impacts and did not sustain any head impacts of this magnitude for the following three days. The cutoff for the 75th percentile of head impact exposure was a day in which an impact load score of 70 was recorded; participants were defined as having experienced “substantial head impact exposure” when an impact load score above this threshold for any practice or game session was recorded (S2 Fig). The 75^th^ percentile was selected because of the large number of less intense impacts and the need for a small number of more intense impacts for analysis of short-term changes.

To analyze microbial population changes across the season while accounting for daily fluctuations, the first three, the middle three, and the last three fecal samples available for each player were analyzed, excluding samples collected after the competition season (S2 Fig). Assumptions of repeated-measures ANOVA, such as Mauchly’s sphericity and assumptions of normality, were not always satisfied. Therefore, Friedman’s Chi-Square Rank Test was consistently used for repeated measures to identify statistically significant differences between the diversity metrics within 72 hours of impact (in increments of 24 hours) and throughout the season, using early, middle, and late as our time points. For significant results from Friedman’s Chi-Square Rank Tests, Nemenyi’s post hoc pairwise testing was performed.

### Data analysis – Mixed effects linear models

Mixed effects models were used to identify linear relationships between the clinical factors measured (S1 Table) and the head impact load, as well as various microbial metrics, including alpha and beta diversity, and the relative abundances of microbial orders, families, genera, and species. A random intercept was used to account for each player, and models were fitted using the lmerTest package in R (version 4.3.1) [51] and adjusted for multiple testing using Benjamini-Hochberg adjustment [52]. The same confounding variables were used for each diversity metric and taxon of interest. However, head impact load was analyzed with 24- and 48-hour offsets to identify time-dependent correlations between head impact and changes in the gut microbiome. Statistical power was computed using the simr package in R. Results from all model runs are provided in Supplementary File 4 (S4 File).

### Data analysis – Principal coordinate analysis

To identify and visualize general patterns in the samples collected across the season, a distance matrix was created using Bray-Curtis dissimilarities based on species level information. The principal coordinates of this distance matrix were computed and plotted as the first two axes to visualize clustering by player. A circle was plotted around each player’s data points using the mean of the centroid as the center and the maximum Euclidean distance between two points within the same player as the radius of the circle.

## Results

### Data collection overview, participant characteristics, and head impact exposure

**Fig 2:**
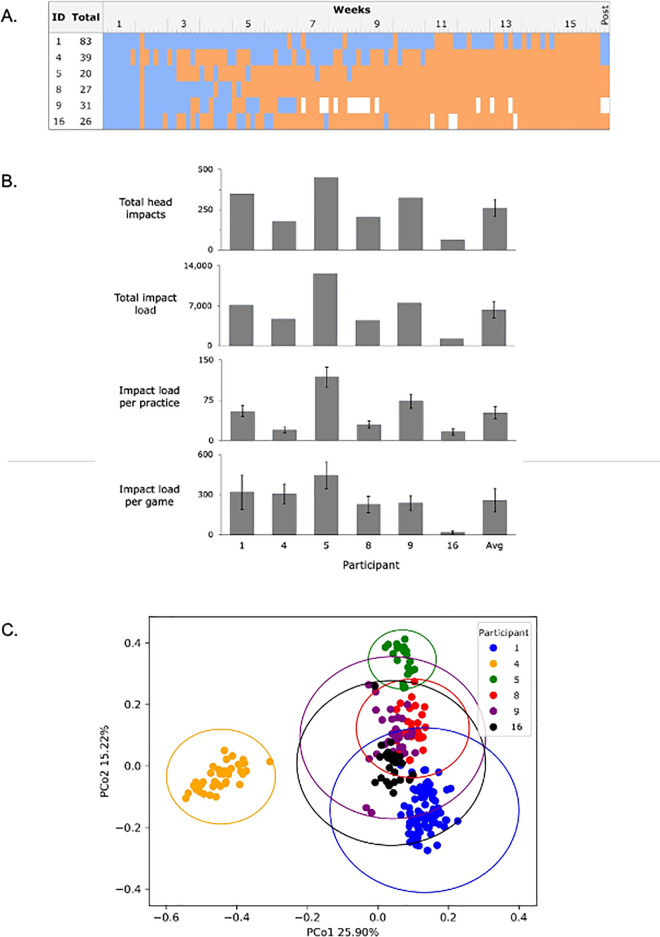
Study participants exhibit variability in head impact frequency and intensity, as well as in fecal microbiome composition. (a) *Availability of fecal samples by participant*. The days on which samples were collected and those on which they were not are marked in blue and orange, respectively. White boxes indicate that the sample collected on this date was excluded due to oral antibiotic use. The final two samples from participants 1, 4, and 9 were collected 10-21 days after the season’s final game. **(b)** *Head impacts and head impact loads sustained by participants*. Error bars represent the standard error of the mean. See Methods for data collection and Supplementary Table 2 (S2 Table) for exact values. **(c)** *Beta diversity analysis of the gut microbiota from all samples used in the study*. The scatter plot displays a principal coordinate analysis (PCoA) with data points color-coded by the participant. Each participant’s data is enclosed within its own ellipsoid.

The consistency of fecal sample collection, questionnaire completion, and head impact exposure varied considerably across the season and among participants (Figs 2a and 2b; S2 Table, S2 Fig). Participants sustained an average of 261 head impacts (SEM 51.9) and an average impact load score of 6,308.3 (SEM 1,435.2) across the season, which accumulated from an average impact load of 52.0 (SEM 11.8) sustained per practice session and of 260.0 (SEM 86.9) sustained per game (Fig 2b, S2 Fig). Two participants began antibiotic use during the season and fecal samples collected after initiating antibiotic treatment were not included in our analysis (Fig 2a).

To examine the relatedness of overall bacterial microbiome composition across the six participants and 226 samples, we performed a principal coordinates analysis. The resulting scatter plot (Fig 2c) illustrates the distribution of the dataset along the first two principal coordinates. Notably, participants 4 (orange), 5 (green), and 8 (red) show low-variance clusters, indicating that their microbiomes collected at different time points are similar along the first two principal coordinates. In contrast, participants 1 (blue), 9 (purple), and 16 (black) show a broader spread, suggesting higher temporal variability within their microbiomes. While the PCoA reveals a distinct clustering of individual participants, we also observed a considerable overlap between some individuals, such as participants 8 (red) and 9 (purple), suggesting similarities in their microbiome compositions.

### Bray-Curtis dissimilarity increases three days following substantial head impact exposure

**Fig 3:**
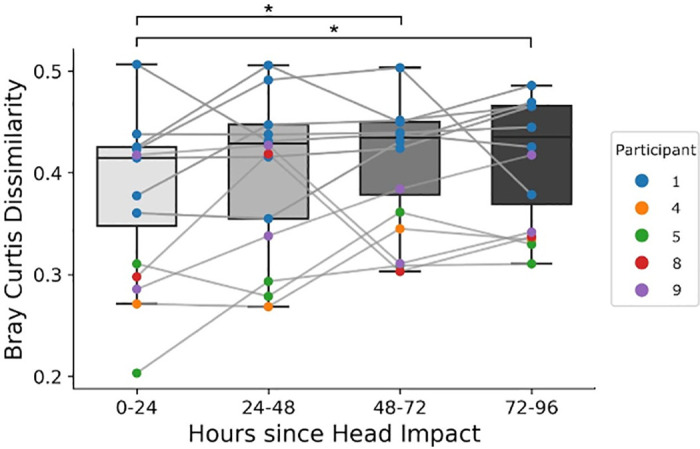
Bray Curtis Dissimilarity increases 48-72 and 72-96 hours following severe head impact exposure. Cluster of fecal samples across four consecutive days isolated such that hour 0 represents a head impact load exposure in the top 75^th^ percentile, followed by 96 consecutive hours in which there was no head impact load exposure in the top 75^th^ percentile. Friedman’s Chi-Square test and Nemenyi’s post hoc pairwise test revealed a significant increase in Bray-Curtis Dissimilarity between 48-72 and 72-96 hours post-impact relative to 0-24 hour (day of impact). Gray lines connect samples from the same data slice. (* indicates p < 0.05).

To examine whether non-concussive head impacts on a single day correlate with changes in gut microbiome composition, we analyzed Bray-Curtis Dissimilarity for each 24 hour period after substantial head impact exposure through 96 hours post-impact. For this analysis, we only considered samples with an impact load in the top 75th percentile (impact load score of 70 or greater) on day one, followed by 3 consecutive days without any head impacts in the 75th percentile (see Methods and S2 Fig). A total of 13 four-day periods met this criterion, with seven coming from Participant 1, one from Participant 4, two from Participant 5, one from Participant 8, two from Participant 9, and none from Participant 16 (Fig 2a). Friedman’s Chi-Squared test on the Bray-Curtis Dissimilarity of the four groups against preseason microbiome composition revealed statistically significant differences in bacterial composition across the four days (𝝌^2^ = 13.02, p = 0.005, df = 13.02) (Fig 3). Nemenyi’s post hoc pairwise test revealed that Bray-Curtis Dissimilarity was significantly higher 48–72 hours (0.434 SEM 0.071, Q57 = 2.85, p = 0.025) and 72–96 hours (0.435 SEM 0.096, Q57 = 3.10, p = 0.012) following substantial head impact exposure when compared to 0–24 hours post (0.415 SEM 0.077) (Fig 3).

### Mixed-effects models accounting for confounding variables confirm connections between head impact exposure and microbial diversity

To determine whether head impact load significantly affects Bray-Curtis Dissimilarity, we fit a mixed-effects linear model accounting for 15 potentially confounding variables that could influence gut microbiome composition (S1 Table). We examined changes in microbial composition over time following head impact exposure by time-shifting the Impact load variable by 0–24, 24–48, or 48–72 hours under three separate models to determine when an effect on Bray-Curtis Dissimilarity might occur relative to head impact exposure or changes in other recorded variables.

Across all three time-shifted models, time (the progression of time across the entire study period; p < 0.001, t = 3.71, df = 194.96), player load (a measure of physical activity intensity during each practice and game; p < 0.001, t = 3.58, df = 195.25), taking non-steroidal anti-inflammatory drugs (NSAIDs) (p = 0.0194, t = 2.36, df = 195.89), and consuming pre-workout energy drinks (p < 0.001, t = 3.55, df = 195.89) were associated with increases in Bray-Curtis Dissimilarity (Table 1). Type 2 stool character (p = 0.0125, t = −2.52, df = 193.28), type 3 stool (p = 0.0083, t = −2.67, df = 193.79), and stress (p = 0.0201, t = −2.35, df = 169.96) were correlated with decreases in Bray-Curtis Dissimilarity. After adjusting for the false discovery rate using the Benjamini-Hochberg adjustment, only the associations between time and Bray-Curtis Dissimilarity (p = 0.0065), player load (p = 0.0065) and consumption of pre-workout energy drinks remained significant (p = 0.0065).

**Table 1:** Bray-Curtis dissimilarity changes in relation to head impacts sustained in the previous 48-72 hours, after adjusting for clinical factors. Significant p-values (< 0.05) are highlighted in dark blue, and marginally significant p-values (< 0.10) are highlighted in light blue. The 48-72 hour time-shifted model is bolded. Only time, player load, and consumption of pre-workout drinks remain significant after the Benjamini-Hochberg p-value correction. “Estimate” indicates the direction and magnitude of the effect on Bray-Curtis Dissimilarity.

Variable	*0-24 hours post*		*24-48 hours post*		*48-72 hours post*	
	Estimate	p (adjusted p)	Estimate	p (adjusted p)	Estimate	p (adjusted p)
*Impact Load*	0.0001538	0.1589 (0.3080)	0.0000334	0.7605 (0.8732)	**0.0002870**	**0.0420 (0.1447)**
*Time (hour)*	0.0000278	0.0202 (0.0846)	0.0000416	0.0011 (0.0104)	**0.0000456**	**0.0003 (0.0065)**
Impact load*time	−0.0000001	0.2291 (0.4177)	0.0000000	0.5217 (0.6738)	**−0.0000002**	**0.1195 (0.2850)**
*Player load*	0.0001332	0.0001 (0.0033)	0.0001294	0.0001 (0.0023)	**0.0001163**	**0.0006 (0.0065)**
*Orthopedic injury*	0.0106443	0.5419 (0.6462)	0.0093275	0.5810 (0.7162)	**0.0103550**	**0.5394 (0.6689)**
*Type 1 – Severe constipation*	−0.0905658	0.0218 (0.0846)	−0.0657809	0.2540 (0.3937)	**−0.0748907**	**0.1899 (0.2896)**
*Type 2 – Minor constipation*	−0.0581556	0.0066 (0.0509)	−0.0564121	0.0092 (0.0476)	**−0.0536494**	**0.0125 (0.0646)**
*Type 3 – Normal*	−0.0286114	0.0337 (0.0950)	−0.0396518	0.0031 (0.0192)	**−0.0353654**	**0.0083 (0.0516)**
*Type 6 – Minor diarrhea*	0.0039469	0.8376 (0.8376)	0.0272812	0.1388 (0.2868)	**0.0252270**	**0.1668 (0.2896)**
*Type 7 – Severe Diarrhea*	0.0893258	0.2563 (0.4210)	0.0930181	0.0576 (0.1984)	**0.0955169**	**0.0515 (0.1597)**
*Less caffeine*	0.0270608	0.2716 (0.4210)	−0.0170889	0.5001 (0.6738)	**−0.0134611**	**0.5898 (0.6874)**
*Normal caffeine*	−0.0208674	0.2599 (0.4210)	−0.0321247	0.1020 (0.2433)	**−0.0254335**	**0.1913 (0.2896)**
*More caffeine*	−0.0325225	0.4381 (0.5659)	−0.0967378	0.0810 (0.2281)	**−0.0828583**	**0.1321 (0.2896)**
*Less NSAIDs*	0.0597370	0.1012 (0.2413)	0.0542994	0.1308 (0.2868)	**0.0507840**	**0.1516 (0.2896)**
*Normal NSAIDs*	0.0320098	0.0297 (0.0919)	0.0349986	0.0138 (0.0611)	**0.0327675**	**0.0194 (0.0778)**
*More NSAIDs*	−0.0629073	0.3335 (0.4700)	0.0298070	0.4582 (0.6738)	**0.0298175**	**0.4525 (0.6100)**
*Less nicotine*	−0.0099746	0.6902 (0.7641)	−0.0333357	0.2236 (0.3648)	**−0.0353403**	**0.1858 (0.2896)**
*Normal nicotine*	−0.0096307	0.6791 (0.7641)	−0.0014977	0.9525 (0.9525)	**−0.0017503**	**0.9436 (0.9922)**
*More nicotine*	0.0502184	0.3070 (0.4532)	0.0691776	0.2091 (0.3601)	**0.0773147**	**0.1539 (0.2896)**
*Pre-workout drink*	0.0617364	0.0034 (0.0354)	0.0859512	0.0001 (0.0023)	**0.0762225**	**0.0005 (0.0065)**
*Number of diet sodas*	−0.0036543	0.8349 (0.8376)	−0.0032012	0.8614 (0.9400)	**−0.0077491**	**0.6696 (0.7414)**
*Alcohol*	0.0019122	0.5417 (0.6462)	0.0003636	0.9025 (0.9400)	**−0.0000051**	**0.9986 (0.9986)**
*Hours of sleep*	0.0202316	0.0184 (0.0846)	0.0152184	0.0790 (0.2281)	**0.0144298**	**0.0908 (0.2559)**
*Sleep quality*	−0.0141333	0.1549 (0.3080)	−0.0065948	0.4967 (0.6738)	**−0.0050543**	**0.5987 (0.6874)**
*Stress rating*	−0.0082775	0.0126 (0.0783)	−0.0075119	0.0267 (0.1036)	**−0.0078570**	**0.0201 (0.0778)**
*Symptom score*	−0.0079122	0.3503 (0.4722)	−0.0044119	0.6007 (0.7162)	**−0.0051181**	**0.5348 (0.6689)**
*Minor illness*	−0.0400530	0.0985 (0.2413)	−0.0363080	0.1676 (0.3247)	**−0.0293673**	**0.2603 (0.3668)**
*Mild illness*	0.0782467	0.0258 (0.0889)	0.0468942	0.1924 (0.3508)	**0.0458310**	**0.1962 (0.2896)**
*Vomitted*	0.0120149	0.7796 (0.8333)	−0.0049708	0.9097 (0.9400)	**0.0021367**	**0.9602 (0.9922)**
*Change in living or dining*	−0.0372360	0.1305 (0.2889)	−0.0383404	0.0903 (0.2333)	**−0.0353493**	**0.1120 (0.2850)**

Impact load exposure 48 to 72 hours prior to fecal sample collection correlated with an increase in Bray-Curtis Dissimilarity (p = 0.0420, t = 2.05, df = 193.13) (Fig 3, Table 1). After adjusting for the false discovery rate using the Benjamini-Hochberg adjustment, the association between impact load and Bray-Curtis Dissimilarity was no longer significant (p = 0.1447). See S4 File for all diversity model runs.

### Gut microbiome composition changes 48–72 hours post head impact

To identify the microbial taxa changes that contribute to changes in Bray-Curtis Dissimilarity 48 to 72 hours following head impact exposure, we performed mixed-effects linear modeling for taxa found to be relevant in the literature (see Methods). Decreases in the relative abundances of the order Coriobacteriales, the family *Prevotellaceae,* and the genus *Prevotella* were significantly correlated with higher impact load (p = 0.0154, t = −2.44, df = 192.89; p = 0.0045, t = −2.87, df = 190.21; p = 0.0035, t = −2.95, df = 190.20; respectively) (Table 2). Increases in the relative abundances of the genus *Ruminococcus* and the order *Verrucomicrobiales* were significantly or marginally correlated with higher impact loads (p = 0.0075, t = 2.70, df = 190.68; p = 0.0776, t = 1.78, df = 195.05; respectively) (Table 2). The interaction effect of impact load and time (impact load*time) was significant or marginally significant for all taxa mentioned (see discussion). After correcting for multiple tests using the Benjamini-Hochberg correction, only the correlations between impact load and the relative abundances of *Prevotellaceae* and *Prevotella* remained significant. Orthopedic injury, caffeine use, pre-workout energy drink consumption, sleep duration, sleep quality, and changes in living or dining circumstances were also significantly correlated with changes in one or more of the five previously mentioned taxa. To assess robustness to the compositional structure of microbiome data, we performed sensitivity analyses using centered log-ratio (CLR) transformation; a subset of associations remained significant, including those for *Prevotellaceae* and *Prevotella*, while others were attenuated (S4 File). In all cases, the direction and magnitude of the effects were consistent with our original observations. These outcomes suggest that the original findings are likely directionally robust but sensitive to compositional bias.

**Table 2:** Changes in relative abundances of Coriobacteriales, Prevotella, Prevotellaceae, Ruminococcus, and Verrucomicrobiales correlate with head impacts sustained in the prior 48-72 hours. Dark blue: p < 0.05; light blue: p < 0.10. “Estimate” indicates direction and magnitude of the correlation between the variable and the relative abundance of the taxa.

Variable	Coriobacteriales		*Prevotellaceae*		*Prevotella*		*Ruminococcus*		*Verrucomicrobiales*	
	Estimate	p (adj p)	Estimate	p (adj p)	Estimate	p (adj p)	Estimate	p (adj p)	Estimate	p (adj p)
***Impact Load***	−0.00005	0.0154 (0.1593)	−0.00004	0.0045 (0.0492)	−0.00003	0.0035 (0.0437)	0.00005	0.0075 (0.0773)	0.00003	0.0775 (0.4803)
***Time (hour)***	0.000001	0.3851 (0.7598)	0.00000	0.9983 (0.9983)	−0.00000	0.9303 (0.9581)	0.000001	0.6000 (0.6889)	0.00000	0.8807 (0.9973)
Impact load*time	0.000000	0.0111 (0.1593)	0.00000	0.0064 (0.0492)	0.00000	0.0056 (0.0437)	0.000000	0.0181 (0.1121)	0.00000	0.0619 (0.4798)
***Player load***	0.000004	0.4167 (0.7598)	0.000001	0.8172 (0.9983)	0.000001	0.6677 (0.9581)	−0.00001	0.0623 (0.2452)	0.000004	0.2230 (0.8640)
***Orthopedic injury***	−0.00394	0.0817 (0.4573)	0.00052	0.7470 (0.9983)	0.00026	0.8477 (0.9581)	0.00068	0.7480 (0.8282)	0.00424	0.0192 (0.2976)
***Severe constipation***	0.00919	0.2285 (0.5449)	0.00008	0.9889 (0.9983)	0.00026	0.9553 (0.9581)	−0.01080	0.1305 (0.3593)	0.00226	0.7095 (0.9973)
***Minor constipation***	0.00474	0.0968 (0.4573)	−0.00016	0.9380 (0.9983)	0.00028	0.8696 (0.9581)	0.0018	0.4987 (0.6184)	−0.0019	0.4015 (0.9888)
***Normal stool***	0.00258	0.1466 (0.5048)	0.00020	0.8773 (0.9983)	0.00022	0.8389 (0.9581)	0.00237	0.1540 (0.3672)	−0.00065	0.6446 (0.9973)
***Minor diarrhea***	0.00399	0.1033 (0.4573)	0.00329	0.0621 (0.3208)	0.00225	0.1326 (0.5139)	0.00147	0.5193 (0.6191)	−0.00108	0.5791 (0.9973)
***Severe Diarrhea***	−0.00528	0.4977 (0.8572)	−0.00194	0.7300 (0.9983)	−0.00215	0.6523 (0.9581)	0.00091	0.9016 (0.9317)	−0.00004	0.9949 (0.9973)
***Less caffeine***	0.00078	0.8149 (0.9025)	0.00419	0.0930 (0.4117)	0.00453	0.0324 (0.1676)	−0.00436	0.1775 (0.3931)	0.00001	0.9973 (0.9973)
***Normal caffeine***	0.00017	0.9493 (0.9493)	0.00195	0.3249 (0.8392)	0.00202	0.2308 (0.6504)	−0.0019	0.4597 (0.6084)	0.00019	0.9299 (0.9973)
***More caffeine***	−0.00347	0.6367 (0.8972)	0.00254	0.6343 (0.9983)	0.00277	0.5402 (0.9581)	0.00604	0.3843 (0.5957)	0.00068	0.9074 (0.9973)
***Less NSAIDs***	−0.00632	0.1824 (0.5141)	0.00016	0.9615 (0.9983)	0.0005	0.8634 (0.9581)	0.00038	0.9322 (0.9322)	0.00111	0.7687 (0.9973)
***Normal NSAIDs***	−0.00160	0.3947 (0.7598)	−0.00071	0.6021 (0.9983)	−0.00031	0.7869 (0.9581)	0.00179	0.3128 (0.5335)	−0.00143	0.3410 (0.9888)
***More NSAIDs***	−0.00255	0.6303 (0.8972)	−0.00309	0.4190 (0.8659)	−0.00247	0.4463 (0.9581)	0.00601	0.2277 (0.4705)	−0.00248	0.5569 (0.9973)
***Less nicotine***	−0.00073	0.8395 (0.9025)	−0.00395	0.1524 (0.5907)	−0.00323	0.1676 (0.5196)	−0.00585	0.1007 (0.3470)	−0.00278	0.3494 (0.9888)
***Normal nicotine***	−0.00114	0.7330 (0.9025)	−0.00223	0.4074 (0.8659)	−0.00228	0.3195 (0.8254)	−0.00549	0.1134 (0.3515)	−0.00056	0.8442 (0.9973)
***More nicotine***	−0.00895	0.2166 (0.5449)	−0.00074	0.8872 (0.9983)	−0.00094	0.8319 (0.9581)	−0.00489	0.4710 (0.6084)	−0.00439	0.4466 (0.9888)
***Pre-workout drink***	−0.00057	0.8443 (0.9025)	0.00964	0.0000 (0.0002)	0.00752	0.0000 (0.0011)	0.00685	0.0129 (0.1003)	−0.00004	0.9861 (0.9973)
***Number of diet sodas***	−0.00132	0.5868 (0.8972)	−.000004	0.9983 (0.9983)	0.00008	0.9581 (0.9581)	−0.00051	0.8217 (0.8783)	−0.00026	0.8943 (0.9973)
***Alcohol***	0.00069	0.0832 (0.4573)	0.00003	0.9212 (0.9983)	0.00008	0.7461 (0.9581)	0.00030	0.4112 (0.5991)	0.00026	0.4057 (0.9888)
***Hours of sleep***	−0.00176	0.1254 (0.4858)	0.00188	0.0275 (0.1702)	0.0017	0.0190 (0.1175)	−0.00337	0.0024 (0.0370)	0.00075	0.4162 (0.9888)
***Sleep quality***	−0.00042	0.7443 (0.9025)	−0.00103	0.2762 (0.7783)	−0.00052	0.5140 (0.9581)	0.00143	0.2433 (0.4713)	−0.00212	0.0419 (0.4329)
***Stress rating***	−0.00060	0.1811 (0.5141)	−0.00043	0.2049 (0.7057)	−0.00052	0.0718 (0.3178)	−0.00035	0.4252 (0.5991)	−0.00053	0.1470 (0.6511)
***Symptom score***	0.00065	0.5566 (0.8972)	−0.00026	0.7456 (0.9983)	−0.0001	0.8841 (0.9581)	−0.00153	0.1391 (0.3593)	0.00004	0.9646 (0.9973)
***Minor illness***	−0.00025	0.9446 (0.9493)	−0.00296	0.2511 (0.7783)	−0.00306	0.1626 (0.5196)	0.00332	0.3230 (0.5335)	−0.00119	0.6757 (0.9973)
***Mild illness***	0.00195	0.6812 (0.9025)	−0.0023	0.5061 (0.9805)	0.0007	0.8118 (0.9581)	−0.0044	0.3270 (0.5335)	−0.00565	0.1382 (0.6511)
***Vomitted***	−0.0061	0.2909 (0.6441)	−0.00377	0.3591 (0.8564)	−0.00315	0.3661 (0.8730)	−0.00997	0.0633 (0.2452)	0.00242	0.5941 (0.9973)
***Change living/dining***	0.00074	0.8035 (0.9025)	−0.00593	0.0057 (0.0492)	−0.00507	0.0053 (0.0437)	−0.00560	0.0440 (0.2275)	0.00978	0.0000 (0.0015)

### Bray-Curtis dissimilarity increases across the sample collection period

**Fig 4:**
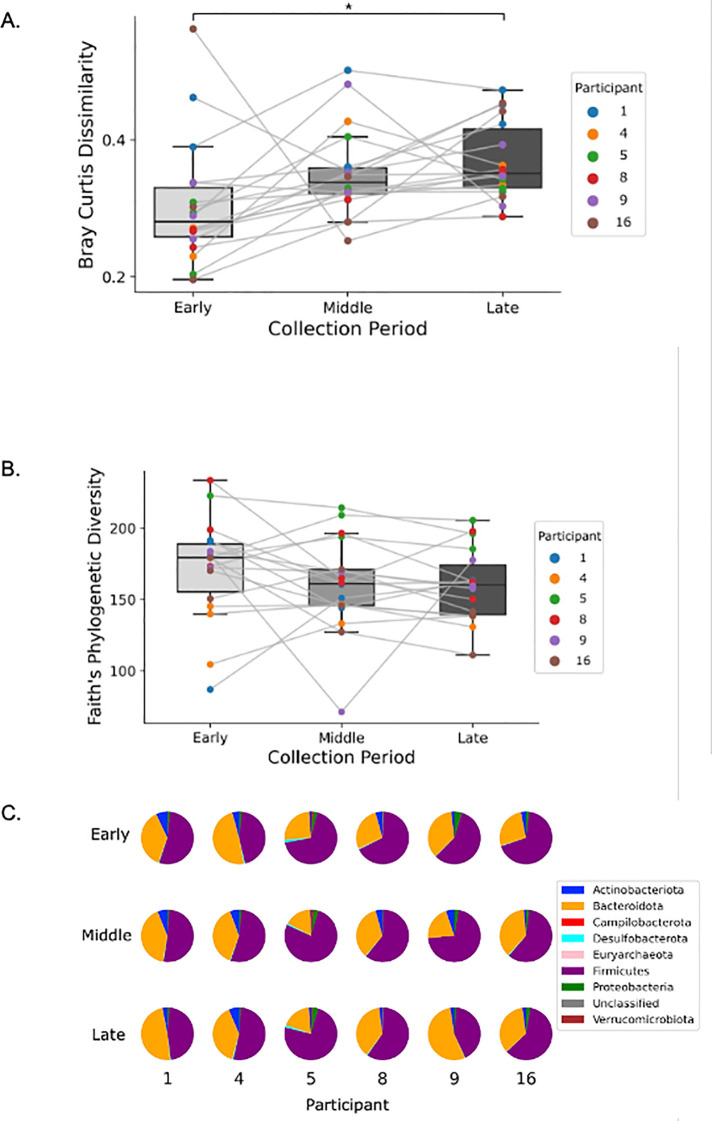
(a) Bray-Curtis Dissimilarity increases across the sample collection period. The first three, the middle three, and the last three fecal samples available for each participant (excluding postseason samples) were used for the early, middle, and late periods, respectively (see methods and S2 Fig). Bray-Curtis Dissimilarity increased from the early to late period (*indicates p < 0.05). (b) Faith’s Phylogenetic Diversity did not change significantly across the sample collection period. Analysis revealed no significant differences in Faith’s Phylogenetic Diversity across the sample collection period for the three time points analyzed. (c) The relative abundance of phyla varies across participants and across the sample collection period. Firmicutes and Bacteroidota were the most abundant phyla for all participants, but their relative abundances varied among participants and across the sample collection period.

To investigate whether the composition of the gut microbiome changed as the season progressed, we examined the differences in microbial diversity between samples collected early, middle, or late in the collection period (see Methods and S2 Fig). When comparing early, middle, and late fecal samples, Friedman’s Chi-Squared test on the Bray-Curtis dissimilarity of the three groups revealed statistically significant differences (𝝌^2^ = 13.44, p = 0.0012, df = 13.44) (Fig 4a). Nemenyi’s post hoc pairwise test revealed that Bray-Curtis dissimilarity was significantly lower early in the collection period (0.280 ± 0.072) than late (0.326 ± 0.048, Q_34_ = 3.71, p = 0.001).

We also investigated changes in alpha diversity across the sample collection period as measured by Faith’s Phylogenetic Diversity (Fig 4b). Friedman’s Chi-Squared test on Faith’s Phylogenetic Diversity revealed no significant differences between any time periods (𝝌^2^ = 3.0, p = 0.2231). While Firmicutes and Bacteroidetes were the most abundant phyla for all participants, their relative abundances varied (Fig 4c). Some variation does occur within individuals across the collection period, with fluctuations in the abundant phyla Firmicutes and Bacteroidetes most visible for players 4, 5, and 9.(4c) (4b)

## Discussion

The present study aimed to determine whether and how the composition of the human fecal microbiome changes in response to nonconcussive head impacts. The rationale for the present study is based on previous literature demonstrating changes in the gut microbiome following mTBI in murine models and the limited available evidence demonstrating similar findings in humans. Our results provide evidence that NHIs sustained in American Football correlate with alterations in the gut microbiome.

The six participants sustained an average of 261 (SEM 51.9) head impacts and an average impact load score of 6,308.25 (SEM 1,435.2) across the season. Of those scores, an average load of 52.0 (SEM 11.8) was sustained per practice session and 260.0 (SEM 86.9) per game (Fig 2b). The number of impacts sustained across the season and per session in our study was consistent with other studies using the same head impact monitoring system on collegiate football players [27].

By isolating incidents with substantial head impact exposure followed by multiple days of low head impact exposure, we observe that Bray-Curtis Dissimilarity increases within 48–72 hours of head impact exposure. We confirmed this head impact effect using mixed-effects linear models accounting for 15 clinically relevant factors, providing additional evidence that NHIs sustained in the past 48–72 hours are associated with changes in microbial diversity. Our models also suggested that decreases in the relative abundances of Coriobacteriales*, Prevotellaceae,* and *Prevotella* and increases in *Ruminococcus* and *Verrucomicrobiales* were correlated with greater head impact exposure. The acute increases in Bray-Curtis dissimilarity we observed following NHIs and the longitudinal increase in dissimilarity we identified across the competition season suggest that an accumulation of NHIs potentially contributes to longitudinal changes in gut microbial diversity.

Our data adds to evidence that mTBIs lead to changes in gut microbial diversity within 72 hrs after head impact [44]. Previous studies have reported that cognitive deficits such as memory impairment, decreased executive function and response time, as well as increases in peripheral neuroinflammatory markers, including S100 calcium-binding protein beta (S100B) and neurofilament light (NfL) [32,53–55], appear to occur before this shift in microbial diversity. Thus, a short-term analysis examining fluctuations in peripheral biomarkers of neuroinflammation and cognitive function following NHI and mTBI could determine whether acute changes in the gut microbiome following brain trauma are linked with the severity of cognitive deficits and pro-inflammatory profiles.

Comparing the alterations in the gut microbiome among human subjects is challenging because individual gut microbiomes can exhibit unique responses to the same input, likely due to the combined effects of host genetics and current microbial composition [56,57]. Nonetheless, our data suggest that the alterations in the relative abundances of specific taxa that we identified match those previously reported in the literature examining brain injury and the microbiome and are functionally relevant. *Prevotellaceae* has been found to decrease in response to TBI in mice, rats, and humans [43,45]. Depletion of *Prevotellaceae* has been associated with increases in pro-inflammatory cytokines and decreased butyrate production in the gut, which has been shown to provide a neuroprotective effect by restoring the blood-brain barrier (BBB) following TBI [58,59]. Additionally, the *Prevotella* genus has been shown to increase the concentration of short-chain fatty acids (SCFAs) in pigs; SCFAs inhibit inflammation and are potentially protective against dementia [60,61]. Yet, certain species of *Prevotella* have been shown to decrease SCFA production and perpetuate intestinal inflammation [62]. Thus, the effect of depletion in *Prevotellaceae* likely depends on species and on the host microbial composition.

Moreover, we found that sustaining a head impact is correlated with an increase in the *Ruminococcus* genus 48–72 hours following the impact; however, in response to brain injuries, other studies have found decreases in the *Ruminococcaceae* and a species of *Ruminococcus* in murine models but long-term increases in the *Ruminococcaceae* family in humans with severe TBI [42–44,47]. The *Ruminococcus* genus and its species are increased in patients with inflammatory bowel disease and have been associated with the production of pro-inflammatory metabolites in Crohn’s disease [63,64]. We also found a marginally significant association between head impact load and increases in *Verrucomicrobiales*. *Listeriaceae* is a family member within this order, which increases in abundance in the gut in response to mTBI in rats [44], and the phylum *Verrucomicrobia* has been reported to increase in response to TBI in humans [43]. The family *Verrucomicrobiaceae* also increases in response to acute inflammation in mice, and the phylum *Verrucomicrobia* was shown to induce inflammation in the colon of rats [65]. The role or effects of these changes remain undetermined.

The changes in the relative abundance of these taxa in response to NHIs and the relevant relationships between the taxonomic shifts and inflammatory states reported in the literature suggest that NHIs may nudge the gut microbiome towards an inflammation-promoting state that could contribute to longer-term neurological consequences. However, the inconsistency of the trends in taxonomic changes across the literature and between organisms indicates that future explorations across a more diverse population are necessary to determine if these taxonomic changes following exposure to NHIs are consistent and relevant. To determine if these changes in taxonomy are linked to inflammatory traits, these studies might also measure SCFA in stool and inflammatory biomarkers in serum before and after exposure to NHIs.

While our observation that Bray-Curtis dissimilarity increases across the season provides evidence for a link between microbiome changes and the accumulation of non-concussive head impacts over the course of a football season, many other variables could also be influencing the gut microbiome during this time period. Factors including diet, workout intensity, health status, sleep, stress, and others may also influence the microbiome composition. The mathematical modeling of our data (S4 File) provides further evidence for a link between NHIs and changes in microbiome composition, even in the context of these other variables. Notably, the interaction effect between head impact load and time (“impact load*time”) was significant in the models for all of the aforementioned taxa (Table 2). The interaction effect may indicate changes in response to head impacts that depend on the host’s current state or changes specific to that host (note the uneven distribution of sample collections between participants over time; see Fig 2a and S2 Fig). This suggests that in-depth, longitudinal analyses of individual microbiomes may be more informative for determining the relationships between specific factors and taxa than group-wide or population studies, particularly when studying humans with distinct microbial profiles.

The mixed-effects linear model also demonstrated potential effects of other factors on the microbiome, including correlations between time (across the entire study period), player load (a measure of physical activity during each practice and game), NSAID use, and consumption of pre-workout energy drinks and increasing dissimilarity in gut microbial composition from the start of the season. The correlation between time and increasing microbiome dissimilarity between players might be explained by other lifestyle changes associated with athletic competition seasons that were not accounted for by the limited survey methods [56]. Similarly, exercise intensity, NSAID use, and ingredients commonly found in pre-workout supplements have been shown to change the diversity and composition of the gut microbiome in humans [66–68]. Reporting “less than normal” and “more than normal” NSAID use was not significantly correlated with Bray-Curtis Dissimilarity (Table 1), likely because participants sparsely reported this behavior (S1 Table).

We find that changes in stress ratings are associated with lower Bray-Curtis dissimilarity compared to the baseline samples, possibly due to the higher reported levels of stress at the beginning of preseason training camp in this study and generally across collegiate athletes [69]. Given that collegiate athletes partake in cyclical behaviors due to the weekly practice and competition cycles, further investigation might include an in-depth analysis of dietary behaviors, activity patterns (outside of those for the sport), and other behaviors to confirm that the associations between NHIs and gut microbiome changes are not due to other confounding behavioral or environmental factors. Moreover, an analysis with additional dependent variables, such as serum levels of inflammatory cytokines and cognitive assessments, might identify other factors beyond brain injury contributing to chronic inflammation and long-term cognitive deficits among American football players.

Although we observed five taxa that responded acutely to NHIs, we did not identify any significant changes in their relative abundances over the collection period. Additionally, we did not detect significant changes in alpha diversity across this period (Fig 4b). The small cohort size and the relatively light impact load burden that Participants 4, 8, and 16 experienced throughout the season could contribute to this lack of effect. However, a visual downward trend in alpha diversity is visible across the collection period, suggesting a trend toward dysbiosis (Fig 4b). A larger sample size is needed to confirm this downward trend and its potential association with NHIs sustained across the season.

An increase in Bray-Curtis Dissimilarity was observed across the season (Fig 4a), which, due to the significant association between Bray-Curtis Dissimilarity and head impact load (Table 1), suggests a potential cumulative effect of NHIs leading to long-term alterations in the gut microbiome. A larger cohort is needed to confirm and characterize this effect. However, the accumulation of NHIs leading to long-term alterations in the gut microbiome could have implications for the chronic inflammation associated with repeated mTBI, which may be linked to short-term disability, neurodegeneration, and early cognitive decline [15–17].

This study, to our knowledge, provides the first evidence for a link between non-concussive head impacts and microbiome composition, both in the short- and long-term. This work provides and important foundation for future studies and a dataset with over 225 individual microbiome samples and metadata sources for further analysis. However, this initial, small-scale study also has considerable limitations. Without a control group and random assignment, this observational study cannot support causal inference; findings should be interpreted as correlational. Mixed-effects models are appropriate for these data, but establishing causality would require a randomized or stronger quasi-experimental design. Furthermore, many of the measures of confounding factors are self-reported. Due to the lack of survey data for a few microbiome samples, we imputed several survey entries, which we recognize could add a small amount of bias. Additionally, we selected a cut-off of 70g as a substantial head impact for our analysis of the short-term effects of non-concussive head impacts to allow for analysis of time periods over which a response may occur. Ideally, the cut-off level is chosen to balance sensitivity and interpretability: it yields enough above-threshold events to analyze, while still allowing us to identify many time slices where an above-threshold impact is followed by multiple days of no impacts or below-threshold impacts. Future studies would use a physiologically-relevant force measure or a larger dataset that would allow analysis of the effects of impacts at other g-forces. Finally, the small sample size and narrow demographic limit the generalizability of the findings. Despite the large number of total samples collected and analyzed, the small number of participants resulted in a power of less than 3% at the 95% confidence level for the mixed-effects modeling. Despite the low statistical power, our models yielded statistical significance for several taxonomic groups. Consistent with this limitation, several associations identified using relative abundance analyses were attenuated following centered log-ratio (CLR) transformation. As a result, we believe the current evidence prompts deeper investigation. Future studies should include a larger sample size with increased numbers of participants, longer longitudinal data, and a comparable control group. In addition, future research should effectively monitor relevant confounding factors, include additional outcome measures, such as assessments of inflammatory biomarkers and cognition, and include female participants, as they respond differently to mTBIs [70].

## Conclusions

To the best of our knowledge, this study provides the first evidence that non-concussive head impacts correlate with both short- and long-term changes in the diversity and composition of the gut microbiome. We observe that a statistically significant change in the overall microbiome occurs within three days after an NHI and that five microbial taxa previously identified as differentially abundant after a TBI or in patients with neurological disorders show changes in abundance. Statistical analysis confirms the correlation between head impacts and these changes, even after controlling for other clinical and behavioral variables. We also observe changes in microbial composition across portions of an American football season, even in the absence of diagnosed mTBIs. This study highlights the need for further research into the effects of both individual non-concussive head impacts and the cumulative effects of repeated impacts across a competition period.

## Supporting information

S1 Fig Verification of control samples. 16S amplification and DNA sequence analysis were performed on five non-fecal samples as controls for taxonomic analysis. The left panel shows the distribution of phyla in the preseason baseline sample from each of the six participants. The right panel shows the distribution of phyla across the five control samples. The internal mock, ATTC, is the ABRF-MGRG 10-strain even mix MSA-3001 (ATCC, Gaithersburg, MD, USA). There were two negative controls: N1 was a blank sample that went through the DNA extraction process, and N2 was just the stock solution that the DNA was dissolved in. The taxonomic profiles of these two samples were similar to those of all six participants. Positive controls (P1 and P2) consisted of 100% *E. coli.*
(TIFF)

S2 Fig Sample collection and head impact overview. Each vertical bar represents an individual date across the collection period. The days on which samples were collected are colored orange or blue. The samples used in the longitudinal analysis are colored blue (see Methods and Fig 4). The dashed green line indicates the threshold for “substantial head impact exposure” (see Methods). Samples outlined in red indicate the starting point (0–24 hours post substantial head impact exposure) for the analysis in Fig 3 (see methods).
(TIFF)

S1 Table Clinical factor descriptions, responses, and exclusions. A total of 30 factors were monitored throughout the study; 28 were obtained from survey data, and 2 were acquired from on-field devices. 12 factors acquired from survey data were excluded due to low reporting or errors in reporting methods. Three dietary factors were excluded due to the flows in the collection methods and the homogeneity of responses. See S1 and S2 Files for survey questions and formats.
(TIFF)

S2 Table Baseline participant demographics, medical history, sports information, and data availability. Days since the last concussion and oral antibiotic use are calculated from the first day of fecal sample collection. The total number of head impacts and impact loads sustained were measured throughout the entire season that the study was conducted.
(TIFF)

S1 File Participant background questionnaire. The survey was completed through Google Forms prior to the beginning of the study and of sample collection.
(PDF)

S2 File Fecal sample collection lifestyle questionnaires. The survey was completed by participants through Google Forms each time they collected a fecal sample.
(PDF)

S3 File Literature review of the taxa used in analysis. A review of the concussion and microbiome response was conducted to identify taxa on which to focus in our examination of microbiome changes after non-concussive head impacts. The taxa used in our analysis of bacterial fluctuations after recorded impacts are highlighted in orange.
(PDF)

S4 File All mixed-effects linear models. Linear models examined in our analysis are provided here.
(XLSX)
