# Supplementary figures and images for: Non-concussive head impacts sustained during American football correlate with changes in gut microbiome diversity and composition
Source: PLoS One. 2026 May 6;21(5):e0345651. doi: 10.1371/journal.pone.0345651 (PMC13148679; doi:10.1371/journal.pone.0345651)

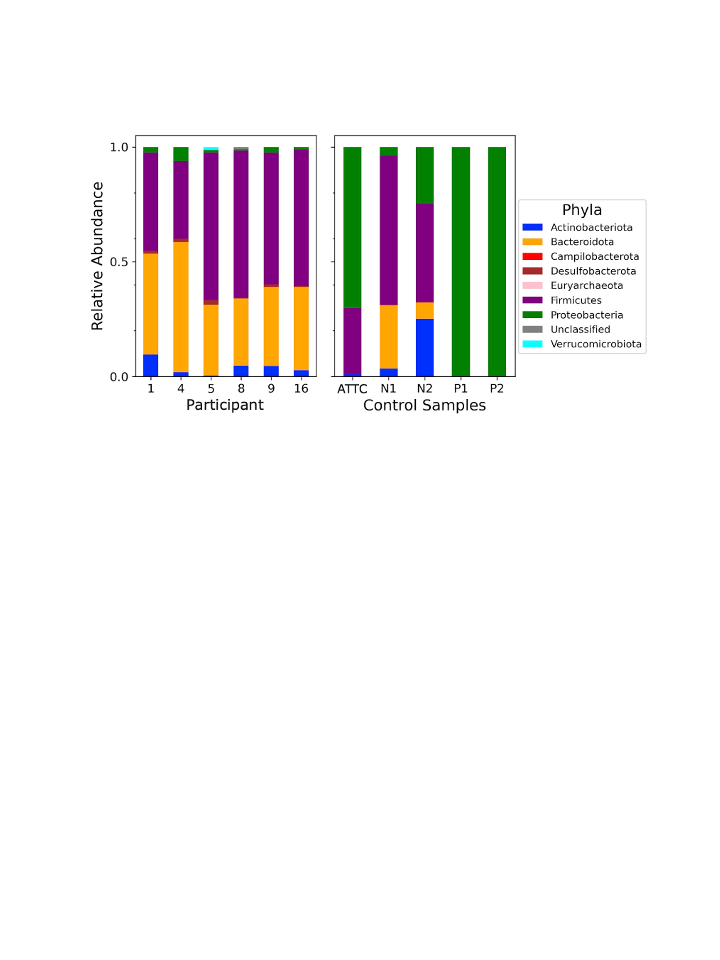

Supplement: S1 Fig — 16S amplification and DNA sequence analysis were performed on five non-fecal samples as controls for taxonomic analysis. The left panel shows the distribution of phyla in the preseason baseline sample from each of the six participants. The right panel shows the distribution of phyla across the five control samples. The internal mock, ATTC, is the ABRF-MGRG 10-strain even mix MSA-3001 (ATCC, Gaithersburg, MD, USA). There were two negative controls: N1 was a blank sample that went through the DNA extraction process, and N2 was just the stock solution that the DNA was dissolved in. The taxonomic profiles of these two samples were similar to those of all six participants. Positive controls (P1 and P2) consisted of 100% E. coli. (TIFF) [file pone.0345651.s001.tiff]

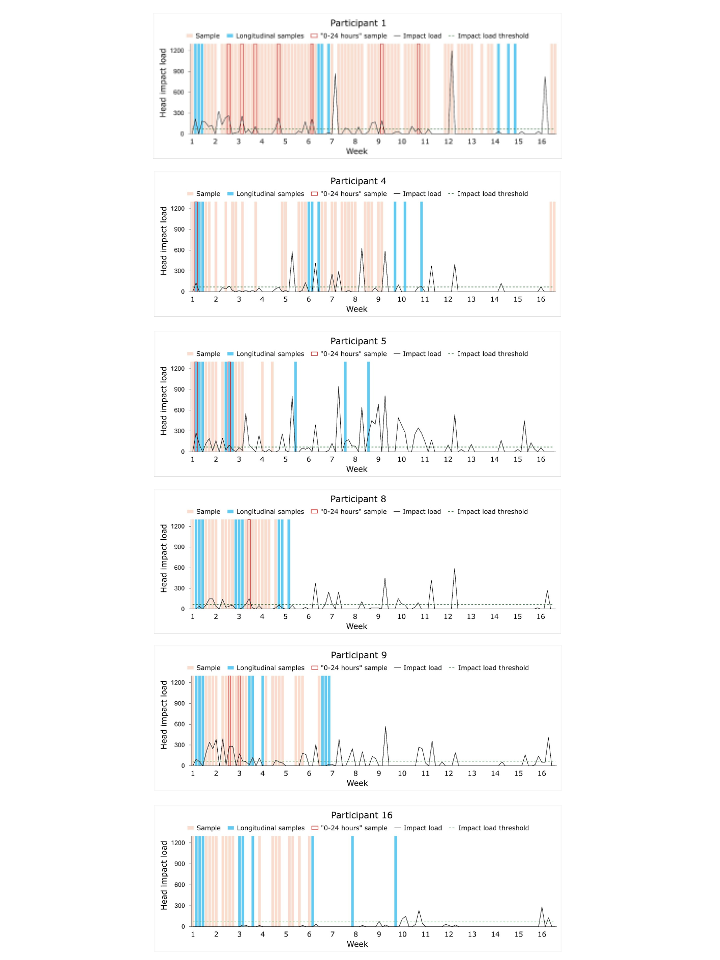

Supplement: S2 Fig — Each vertical bar represents an individual date across the collection period. The days on which samples were collected are colored orange or blue. The samples used in the longitudinal analysis are colored blue (see Methods and Fig 4). The dashed green line indicates the threshold for “substantial head impact exposure” (see Methods). Samples outlined in red indicate the starting point (0–24 hours post substantial head impact exposure) for the analysis in Fig 3 (see methods). (TIFF) [file pone.0345651.s002.tiff]

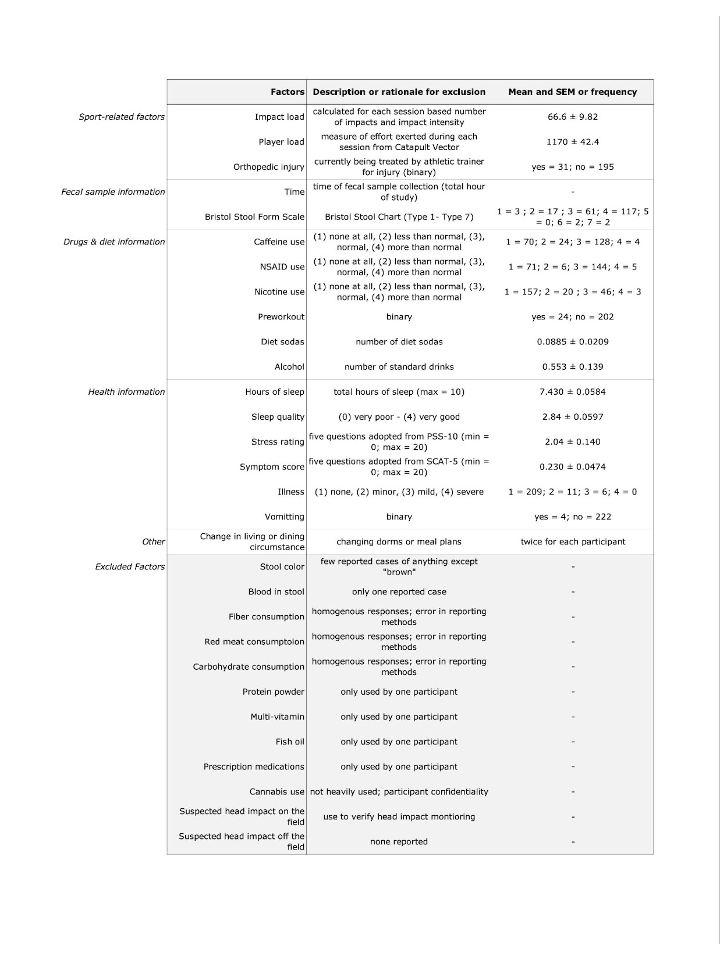

Supplement: S1 Table — A total of 30 factors were monitored throughout the study; 28 were obtained from survey data, and 2 were acquired from on-field devices. 12 factors acquired from survey data were excluded due to low reporting or errors in reporting methods. Three dietary factors were excluded due to the flows in the collection methods and the homogeneity of responses. See S1 and S2 Files for survey questions and formats. (TIFF) [file pone.0345651.s003.tiff]

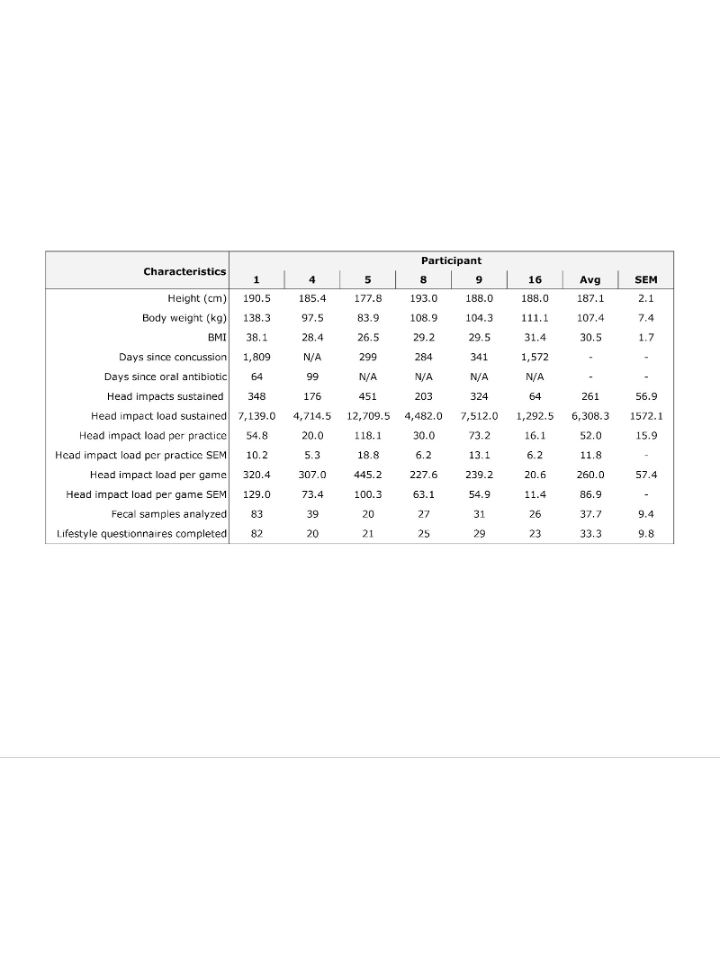

Supplement: S2 Table — Days since the last concussion and oral antibiotic use are calculated from the first day of fecal sample collection. The total number of head impacts and impact loads sustained were measured throughout the entire season that the study was conducted. (TIFF) [file pone.0345651.s004.tiff]
