# Supplementary material for: Non-concussive head impacts sustained during American football correlate with changes in gut microbiome diversity and composition
Source: PLoS One. 2026 May 6;21(5):e0345651. doi: 10.1371/journal.pone.0345651 (PMC13148679; doi:10.1371/journal.pone.0345651)
Supplement: S1 File — The survey was completed through Google Forms prior to the beginning of the study and of sample collection. (PDF) [file pone.0345651.s005.pdf]

Pre-Participation Questionnaire - Head Impacts and The Gut Microbiome

This questionnaire intends to acquire demographic, sport history, and health/medical information before you participate in the study. Please answer all questions as honestly and thoroughly as possible.

This survey should take approximately 20 minutes to complete.

RISKS:
- Some questions could cause the participant distress. Please review your initial consent form for further information.

ALL INFORMATION COLLECTED WILL REMAIN CONFIDENTIAL.

If you have any questions or concerns, please contact ----

* Indicates required question

INFORMED CONSENT

1. After reading the risks associated with this portion of the study, do you still wish to participate? *

Mark only one oval.

- ◯ Yes *Skip to question 2*
- ◯ No

Skip to question 2

PARTICIPATION DECLINE

You have declined to participate in this portion of the study.

If this was a mistake, please press the 'back' button, and change your response to the previous question.

PARTICIPANT ID

Your four-digit participant ID should have been given to you by your survey administrator.

If you have not been given your participant ID or cannot remember it, please contact ---

2. Participant ID *

____

DEMOGRAPHIC INFORMATION

3. Date of Birth

____

Example: January 7, 2019

4. What is your age?

____

5. Which of the following best describes your sex?

Mark only one oval.

- ( ) Male
- ( ) Female
- ( ) Prefer not to specify
- ( ) Other: _______________________________

6. Which of the following best describes your race/ethnicity?

Check all that apply.

- [ ] Asian or Pacific Islander
- [ ] Black or African American
- [ ] Hispanic or Latino
- [ ] Native American or Alaskan Native
- [ ] White or Caucasian
- [ ] Multiracial or Biracial
- [ ] A race/ethnicity not listed here or another way you describe yourself
- [ ] Other: ______________________

SPORT HISTORY

7. At approximately which age did you begin playing full-contact football?

Mark only one oval.

- ◯ younger than 8 years old
- ◯ 8 years old
- ◯ 9 years old
- ◯ 10 years old
- ◯ 11 years old
- ◯ 12 years old
- ◯ 13 years old
- ◯ 14 years old
- ◯ 15 years old
- ◯ 16 years old
- ◯ older than 16 years old

8. What academic year will you be during the fall of 2022?

Mark only one oval.

- Freshman
- Sophomore
- Junior
- Senior
- 5th Year Senior

9. What position do you currently play?

Mark only one oval.

- OL
- TE
- WR
- RB
- QB
- DL
- LB
- DB
- SPEC

HEALTH/MEDICAL INFORMATION - Section I

Reminder: All medical information provided will remain confidential

Concussion History

This includes any and all concussions (inside and outside of football)

10. Have you been diagnosed with a concussion in the past 12 months?

Mark only one oval.

- ( ) Yes
- ( ) No
- ( ) Multiple

11. Including any concussions in the past 12 months, how many total concussions have you been diagnosed with in the past?

Mark only one oval.

- ( ) 0
- ( ) 1
- ( ) 2
- ( ) 3
- ( ) 4
- ( ) 5
- ( ) Greater than 5

12. Please list the approximate date of your MOST RECENT diagnosed concussion (if applicable).

____

Example: January 7, 2019

13. Please list the approximate date of your SECOND MOST RECENT diagnosed concussion (if applicable).

____

Example: January 7, 2019

14. Please list the approximate date of your THIRD MOST RECENT diagnosed concussion (if applicable).

____

Example: January 7, 2019

15. Please list the approximate date of your FOURTH MOST RECENT diagnosed concussion (if applicable).

____

Example: January 7, 2019

16. Please list the approximate date of your FIFTH MOST RECENT diagnosed concussion (if applicable).

____

Example: January 7, 2019

HEALTH/MEDICAL INFORMATION - Section II

REMINDER: All medical information provided will remain confidential

Body Composition

17. Height (inches)

Mark only one oval.

- 4' 8"
- 4' 9"
- 4' 10"
- 4' 11"
- 5' 0"
- 5' 1"
- 5' 2"
- 5' 3"
- 5' 4"
- 5' 5"
- 5' 6"
- 5' 7"
- 5' 8"
- 5' 9"
- 5' 10"
- 5' 11"
- 6' 0"
- 6' 1"
- 6' 2"
- 6' 3"
- 6' 4"
- 6' 5"
- 6' 6"
- 6' 7"
- 6' 8"
- 6' 9"
- 6' 10"
- 6' 11"
- 7' 0"
- 7' 1"

- 7' 2"
- 7' 3"

18. Weight (lbs)

Mark only one oval.

- 90
- 95
- 100
- 105
- 110
- 115
- 120
- 125
- 130
- 135
- 140
- 145
- 150
- 155
- 160
- 165
- 170
- 175
- 180
- 185
- 190
- 195
- 200
- 205
- 210
- 215
- 220
- 225
- 230
- 235

- 240
- 245
- 250
- 255
- 260
- 265
- 270
- 275
- 280
- 285
- 290
- 295
- 300
- 305
- 310
- 315
- 320
- 325
- 330
- 335
- 340
- 345
- 350

Diet/Supplements

19. Do you attempt to partake in any restrictive or exclusionary diets?

Check all that apply.

- [ ] Keto
- [ ] Carinvore
- [ ] Vegetarian
- [ ] Vegan
- [ ] Gluten-free
- [ ] Dairy-free
- [ ] Mediterranean
- [ ] Other: ____________________

20. Do you attempt to eat high amounts of any of the following macromolecules?

Check all that apply.

- [ ] Protein
- [ ] Carbohydrates
- [ ] Fiber
- [ ] Saturated Fats
- [ ] Unsaturated Fats
- [ ] Sugars
- [ ] Other: ____________________

21. Do you attempt to eat low amounts of any of the following macromolecules?

Check all that apply.

- [ ] Protein
- [ ] Carbohydrates
- [ ] Fiber
- [ ] Saturated Fats
- [ ] Unsaturated Fats
- [ ] Sugars
- [ ] Pro-biotics
- [ ] Other: ____________________

22. Do you regularly take any of the following supplements?

Check all that apply.

- [ ] Pro-biotics
- [ ] Fiber Supplements
- [ ] Protein Powder
- [ ] Creatine
- [ ] Pre-workout
- [ ] Multi-vitamin
- [ ] Vitamin D
- [ ] Fish Oil
- [ ] Other: ____________________

Medications and Underlying Medical Conditions

REMINDER: All medical information provided will remain confidential.

23. Have you been prescribed oral antibiotics in the past 12 months (e.g. amoxicillin, doxycycline, penicillin, etc.)?

Mark only one oval.

- ( ) Yes
- ( ) No

24. If you have been prescribed oral antibiotics in the past 12 months, approximately when was your last dose?

____

Example: January 7, 2019

25. If you have been prescribed oral antibiotics in the past 12 months, which oral antibiotics were your prescribed?

Antibacterials			
Class	Name	Indication*	Maximum therapeutic dosage*
Penicillin group	Amoxicillin	AOM, Streptococcal pharyngitis, Pneumonia, Skin infections, UTI, Early or latent syphilis	(500 mg/dose)
	Amoxicillin + clavonic acid		2.4 g amoxicillin/ 600 mg clavulanic acid
	Ampicilline		2-3 g/day
	Penicillin		24 million units/day
	Cloxacillin		4 g/day
Cephalosporins	Cephalexin	LRTI, OM, Skin infection, UTI, Sinusitis, Bronchitis, Cystitis, Pharyngitis, Gastro-enteritis, Gonorrhoea, Lyme disease, Meningitis, Pneumonia, Surgical infection prophylaxis	2 g/day
	Cefixime		400 mg/day
	Ceftriaxone		4 g/day
Macrolides	Azithromycin	RTI, OM, Bronchitis, Pneumonia, Sinusitis, Prophylaxis of surgical infection in bowel surgery, Uncomplicated Genital infections, H pylori regimen	2 g/day (500 mg/dose)
	Erythromycin		4 g/day
	Clarithromycin		1 g/day (500 mg/dose)
Tetracyclines	Doxycycline	Pharyngitis, Sinusitis, or Pneumonia, Non-gonococcal, Urethritis, Conjunctivitis	200 mg/day
	Tetracycline		3 g/day
Quinolones	Ciprofloxacin	Infected bites, sinusitis, OM, Ear, Nose and throat infections, Gastro-enteritis, Gonorrhoea, PID, LRTI, UTI, Skin infection	1 g/day
	Ofloxacin		1.6 g/day
Other antibacterials	Metronidazole	PID, H pylori regimen UTI, RTI, Gastrointestinal tracts infection, Antibiotic associated Colitis, Bacterial vaginosis, Pharyngitis, Pneumonia	4 g/day
	Cotrimaxazole		3.8 g/day
	Clindamycin		1.2-1.8 g/day

*Data were extracted from ***Martindale: The Complete Drug Reference, 37th edition***
Definition of abbreviations: OM: otitis media, AOM: acute otitis media, UTI: urinary tract infection, PID: pelvic inflammatory disease
URTI: upper respiratory tract infection, LRTI: lower respiratory tract infection

Check all that apply.

- [ ] Amoxicillin
- [ ] Ampicilline
- [ ] Penicillin
- [ ] Cloxcillin
- [ ] Cephalexix
- [ ] Cefixime
- [ ] Ceftriaxone
- [ ] Azithromycin
- [ ] Erythromycin
- [ ] Clarifthromycin
- [ ] Doxycucline
- [ ] Tetracycline

- [ ] Ciprofloxacin
- [ ] Ofloxacin
- [ ] Metronidazole
- [ ] Contrimaxazole
- [ ] Clindamycin
- [ ] I am unsure
- [ ] Other: ____________________

26. If you regularly take any prescription medications, please list them here along with the doses and frequency.

____

____

____

____

____

27. If you are currently diagnosed with any underlying medical conditions (orthopedic, psychological, gastrointestinal, cardiac, etc.), please select them here.

Check all that apply.

- [ ] Anxiety
- [ ] Depression
- [ ] Attention Deficit Hyperactive Disorder
- [ ] Autism Spectrum Disorder
- [ ] Schizoaffective Disorder
- [ ] Schizophrenia
- [ ] Bipolar Disorder
- [ ] Dementia or Alzheimers
- [ ] Parkinson's
- [ ] Multiple sclerosis
- [ ] Psoriasis/psoriatic arthritis
- [ ] Migraine Disorders
- [ ] High blood pressure
- [ ] Inflammatory bowel disease
- [ ] Eczema
- [ ] Rosacea
- [ ] Dermatitis Herpetiformis
- [ ] Obesity
- [ ] Type 1 diabetes
- [ ] Type 2 Diabetes
- [ ] Osteoarthritis
- [ ] Celiac disease
- [ ] Pernicious anemia
- [ ] Autoimmune vasculitis
- [ ] Myasthenia gravis
- [ ] Hashimoto’s thyroiditis
- [ ] Sjögren’s syndrome
- [ ] Graves’ disease
- [ ] Addison’s disease
- [ ] Systemic lupus erythematosus
- [ ] Rheumatoid arthritis
- [ ] Other: ______________________

HEALTH/MEDICAL INFORMATION - Section III

REMINDER: All medical information provided will remain confidential.

Over the last 2 weeks, how often have you felt the following?

28. Feeling nervous, anxious, or on edge
(0 = Not at all, 1 = several days, 2 = More than half the days, 3 = Nearly every day)

Mark only one oval.

	0	1	2	3	
Not	◯	◯	◯	◯	Nearly every day

29. Not being able to stop or control worrying
(0 = Not at all, 1 = several days, 2 = More than half the days, 3 = Nearly every day)

Mark only one oval.

	0	1	2	3	
Not	◯	◯	◯	◯	Nearly every day

30. Worrying too much about different things
(0 = Not at all, 1 = several days, 2 = More than half the days, 3 = Nearly every day)

Mark only one oval.

	0	1	2	3	
Not	◯	◯	◯	◯	Nearly every day

31. Trouble relaxing

(0 = Not at all, 1 = several days, 2 = More than half the days, 3 = Nearly every day)

Mark only one oval.

	0	1	2	3	
Not	◯	◯	◯	◯	Nearly every day

32. Being so restless that it is hard to sit still

(0 = Not at all, 1 = several days, 2 = More than half the days, 3 = Nearly every day)

Mark only one oval.

	0	1	2	3	
Not	◯	◯	◯	◯	Nearly every day

33. Becoming easily annoyed or irritable

(0 = Not at all, 1 = several days, 2 = More than half the days, 3 = Nearly every day)

Mark only one oval.

	0	1	2	3	
Not	◯	◯	◯	◯	Nearly every day

34. Feeling afraid as if something awful might happen

(0 = Not at all, 1 = several days, 2 = More than half the days, 3 = Nearly every day)

Mark only one oval.

	0	1	2	3	
Not	◯	◯	◯	◯	Nearly every day

HEALTH/MEDICAL INFORMATION - Section IV

REMINDER: All medical information provided will remain confidential.

Over the last 2 weeks, how often have you felt the following?

35. Little interest or pleasure in doing things
(0 = Not at all, 1 = several days, 2 = More than half the days, 3 = Nearly every day)

Mark only one oval.

	0	1	2	3	
Not	◯	◯	◯	◯	Nearly every day

36. Feeling down, depressed or hopeless
(0 = Not at all, 1 = several days, 2 = More than half the days, 3 = Nearly every day)

Mark only one oval.

	0	1	2	3	
Not	◯	◯	◯	◯	Nearly every day

37. Trouble falling asleep, staying asleep, or sleeping too much
(0 = Not at all, 1 = several days, 2 = More than half the days, 3 = Nearly every day)

Mark only one oval.

	0	1	2	3	
Not	◯	◯	◯	◯	Nearly every day

38. Feeling tired or having little energy

(0 = Not at all, 1 = several days, 2 = More than half the days, 3 = Nearly every day)

Mark only one oval.

	0	1	2	3	
Not	◯	◯	◯	◯	Nearly every day

39. Poor appetite or overeating

(0 = Not at all, 1 = several days, 2 = More than half the days, 3 = Nearly every day)

Mark only one oval.

	0	1	2	3	
Not	◯	◯	◯	◯	Nearly every day

40. Feeling bad about yourself - or that you're a failure or have let yourself or your family down

(0 = Not at all, 1 = several days, 2 = More than half the days, 3 = Nearly every day)

Mark only one oval.

	0	1	2	3	
Not	◯	◯	◯	◯	Nearly every day

41. Trouble concentrating on things, such as reading the newspaper or watching television

(0 = Not at all, 1 = several days, 2 = More than half the days, 3 = Nearly every day)

Mark only one oval.

	0	1	2	3	
Not	◯	◯	◯	◯	Nearly every day

42. Moving or speaking so slowly that other people could have noticed. Or, the opposite - being so fidgety or restless that you have been moving around a lot more than usual

(0 = Not at all, 1 = several days, 2 = More than half the days, 3 = Nearly every day)

Mark only one oval.

	0	1	2	3	
Not	◯	◯	◯	◯	Nearly every day

43. Thoughts that you would be better off dead or of hurting yourself in some way

(0 = Not at all, 1 = several days, 2 = More than half the days, 3 = Nearly every day)

Mark only one oval.

	0	1	2	3	
Not	◯	◯	◯	◯	Nearly every day

HEALTH/MEDICAL INFORMATION - Section V

REMINDER: All medical information provided will remain confidential.

Over the last 2 weeks, how often have you experienced the following?

44. Stomach or bowel problems
(0 = Not at all, 1 = A little bit, 2 = Somewhat, 3 = quite a bit, 4 = very much)

Mark only one oval.

	0	1	2	3	4	
Not	◯	◯	◯	◯	◯	Very much

45. Back pain
(0 = Not at all, 1 = A little bit, 2 = Somewhat, 3 = quite a bit, 4 = very much)

Mark only one oval.

	0	1	2	3	4	
Not	◯	◯	◯	◯	◯	Very much

46. Pain in your arms, legs, or joints
(0 = Not at all, 1 = A little bit, 2 = Somewhat, 3 = quite a bit, 4 = very much)

Mark only one oval.

	0	1	2	3	4	
Not	◯	◯	◯	◯	◯	Very much

47. Headaches
(0 = Not at all, 1 = A little bit, 2 = Somewhat, 3 = quite a bit, 4 = very much)

Mark only one oval.

	0	1	2	3	4	
Not	◯	◯	◯	◯	◯	Very much

48. Chest pain or shortness of breath

(0 = Not at all, 1 = A little bit, 2 = Somewhat, 3 = quite a bit, 4 = very much)

Mark only one oval.

	0	1	2	3	4	
Not	◯	◯	◯	◯	◯	Very much

49. Dizziness

(0 = Not at all, 1 = A little bit, 2 = Somewhat, 3 = quite a bit, 4 = very much)

Mark only one oval.

	0	1	2	3	4	
Not	◯	◯	◯	◯	◯	Very much

50. Feeling tired or having low energy

(0 = Not at all, 1 = A little bit, 2 = Somewhat, 3 = quite a bit, 4 = very much)

Mark only one oval.

	0	1	2	3	4	
Not	◯	◯	◯	◯	◯	Very much

51. Trouble sleeping

(0 = Not at all, 1 = A little bit, 2 = Somewhat, 3 = quite a bit, 4 = very much)

Mark only one oval.

	0	1	2	3	4	
Not	◯	◯	◯	◯	◯	Very much

HEALTH/MEDICAL INFORMATION - Section VI

Respond to the following questions on a scale of 0-4

52. How often do you have trouble wrapping up the final details of a project, once the challenging parts have been done?
(0 = never, 1 = rarely, 2 = sometimes, 3 = often, 4 = very often)

Mark only one oval.

	0	1	2	3	4	
Not	◯	◯	◯	◯	◯	Very often

53. How often do you have difficulty getting things in order when you have to do a task that requires organization?
(0 = never, 1 = rarely, 2 = sometimes, 3 = often, 4 = very often)

Mark only one oval.

	0	1	2	3	4	
Not	◯	◯	◯	◯	◯	Very often

54. How often do you have problems remembering appointments or obligations?
(0 = never, 1 = rarely, 2 = sometimes, 3 = often, 4 = very often)

Mark only one oval.

	0	1	2	3	4	
Not	◯	◯	◯	◯	◯	Very often

55. When you have a task that requires a lot of thought, how often do you avoid or delay getting started?
(0 = never, 1 = rarely, 2 = sometimes, 3 = often, 4 = very often)

Mark only one oval.

	0	1	2	3	4	
Not	◯	◯	◯	◯	◯	Very often

56. How often do you fidget or squirm with your hands or feet when you have to sit down for a long time?
(0 = never, 1 = rarely, 2 = sometimes, 3 = often, 4 = very often)

Mark only one oval.

	0	1	2	3	4	
Not	◯	◯	◯	◯	◯	Very often

57. How often do you feel overly active and compelled to do things, like you were driven by a motor?
(0 = never, 1 = rarely, 2 = sometimes, 3 = often, 4 = very often)

Mark only one oval.

	0	1	2	3	4	
Not	◯	◯	◯	◯	◯	Very often

HEALTH/MEDICAL INFORMATION - Section VII

REMINDER: All medical information provided will remain confidential.

Please answer the following questions about ALL of the headaches you have had over the last 3 months.

Select your answer on the scale below each question. Select zero if you did not have the activity in the last 3 months. Select 10 if your answer is 10 or greater.

58. On how many days in the last 3 months did you miss work or school because of headaches?

Select your answer on the scale below each question. Select zero if you did not have the activity in the last 3 months. Select 10 if your answer is 10 or greater.

Mark only one oval.

0	1	2	3	4	5	6	7	8	9	10
◯	◯	◯	◯	◯	◯	◯	◯	◯	◯	◯

59. How many days in the last 3 months was your productivity at work or school reduced by half or more because of headaches? (Do not include days you counted in question 1 where you missed work or school.)

Select your answer on the scale below each question. Select zero if you did not have the activity in the last 3 months. Select 10 if your answer is 10 or greater.

Mark only one oval.

0	1	2	3	4	5	6	7	8	9	10
◯	◯	◯	◯	◯	◯	◯	◯	◯	◯	◯

60. On how many days in the last 3 months did you not do household work (such as housework, home repairs and maintenance, shopping, caring for children and relatives) because of headaches?

Select your answer on the scale below each question. Select zero if you did not have the activity in the last 3 months. Select 10 if your answer is 10 or greater.

Mark only one oval.

0	1	2	3	4	5	6	7	8	9	10
◯	◯	◯	◯	◯	◯	◯	◯	◯	◯	◯

61. How many days in the last 3 months was your productivity in household work reduced by half of more because of headaches? (Do not include days you counted in question 3 where you did not do household work.)

Select your answer on the scale below each question. Select zero if you did not have the activity in the last 3 months. Select 10 if your answer is 10 or greater.

Mark only one oval.

0	1	2	3	4	5	6	7	8	9	10
◯	◯	◯	◯	◯	◯	◯	◯	◯	◯	◯

62. On how many days in the last 3 months did you miss family, social or leisure activities because of headaches?

Select your answer on the scale below each question. Select zero if you did not have the activity in the last 3 months. Select 10 if your answer is 10 or greater.

Mark only one oval.

0	1	2	3	4	5	6	7	8	9	10
◯	◯	◯	◯	◯	◯	◯	◯	◯	◯	◯

QUESTIONNAIRE SUBMISSION - PRESS SUBMIT

Thank you for completing the pre-study questionnaire. Please press submit to complete the questionnaire.

This content is neither created nor endorsed by Google.

Google Forms
