# Supplementary material for: Non-concussive head impacts sustained during American football correlate with changes in gut microbiome diversity and composition
Source: PLoS One. 2026 May 6;21(5):e0345651. doi: 10.1371/journal.pone.0345651 (PMC13148679; doi:10.1371/journal.pone.0345651)
Supplement: S2 File — The survey was completed by participants through Google Forms each time they collected a fecal sample. (PDF) [file pone.0345651.s006.pdf]

Daily Questionnaire - Head Impacts and The Gut Microbiome

This is a daily that will assess certain fluctuating variables that might impact with the gut microbiome changes or the effects head impacts. Please answer all questions as honestly and thoroughly as possible.

Please complete this questionnaire directly AFTER you collect your stool sample.

This questionnaire should should take less than 5 minutes to complete.

RISKS:
- Some questions could cause the participant distress. Please review your initial consent form for further information.

ALL INFORMATION COLLECTED WILL REMAIN CONFIDENTIAL.

If you have any questions or concerns, please contact ---

* Indicates required question

INFORMED CONSENT

1. After reading the risks associated with this portion of the study, do you still wish to participate? *

Mark only one oval.

- Yes *Skip to question 2*
- No

Skip to question 2

PARTICIPATION DECLINE

You have declined to participate in this portion of the study.

If this was a mistake, please press the 'back' button, and change your response to the previous question.

PARTICIPANT ID

Your four-digit participant ID should have been given to you by your survey administrator.

If you have not been given your participant ID or cannot remember it, please contact ---

2. Participant ID *

____

STOOL SAMPLE INFORMATION

3. Were you able to collect a stool sample today? *

Mark only one oval.

- ( ) Yes
- ( ) No

4. At what time did you produce your sample? *

____

Example: 8:30 AM

5. Which image most accurately represents the consistency of the stool from your sample today? *

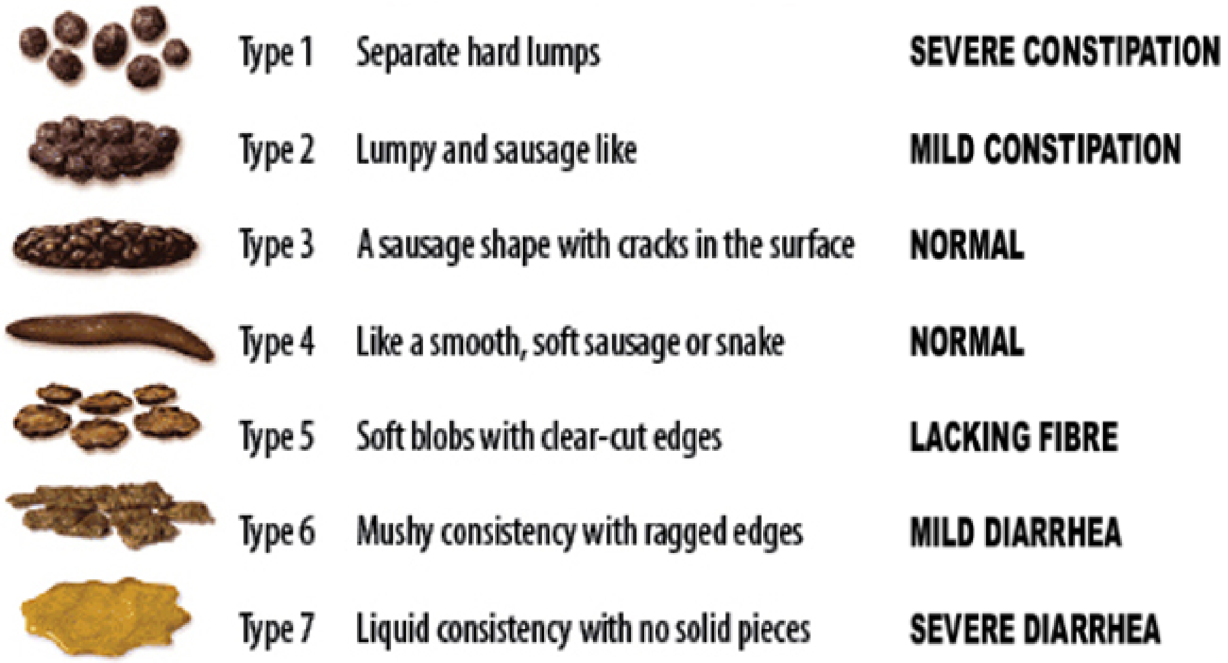

Mark only one oval.

- ⬭ Type 1
- ⬭ Type 2
- ⬭ Type 3
- ⬭ Type 4
- ⬭ Type 5
- ⬭ Type 6
- ⬭ Type 7
- ⬭ I did not collect a sample today

6. What color was your stool?

Mark only one oval.

- ◯ Red
- ◯ Black
- ◯ Brown
- ◯ Slightly Green
- ◯ Very Green

7. Was there any blood in your stool or on the toilet paper after you wiped?

Mark only one oval.

- ◯ Yes
- ◯ No
- ◯ I'm not sure

HEALTH AND WELLNESS

REMINDER: All medical information provided will remain confidential.

8. In the past 24 hours, how often have you been upset because of something that happened unexpectedly?

0 - never; 1 - almost never; 2 - sometimes; 3 - fairly often; 4 - very often

Mark only one oval.

	0	1	2	3	4	
Nev	◯	◯	◯	◯	◯	Very often

9. In the past 24 hours, how often have you felt nervous and stressed?

0 - never; 1 - almost never; 2 - sometimes; 3 - fairly often; 4 - very often

Mark only one oval.

	0	1	2	3	4	
Nev	◯	◯	◯	◯	◯	Very often

10. In the past 24 hours, how often have you felt confident about your ability to handle your personal problems?

0 - never; 1 - almost never; 2 - sometimes; 3 - fairly often; 4 - very often

Mark only one oval.

	0	1	2	3	4	
Nev	◯	◯	◯	◯	◯	Very often

11. In the past 24 hours, how often have you felt that you could not cope with everything you had to do?

0 - never; 1 - almost never; 2 - sometimes; 3 - fairly often; 4 - very often

Mark only one oval.

	0	1	2	3	4	
Nev	◯	◯	◯	◯	◯	Very often

12. In the past 24 hours, how often have you felt like you were on top of things?

0 - never; 1 - almost never; 2 - sometimes; 3 - fairly often; 4 - very often

Mark only one oval.

	0	1	2	3	4	
Nev	◯	◯	◯	◯	◯	Very often

13. In the past 24 hours, approximately how many hours have you slept?

Select 10 hours if you slept more than 10 hours.

Mark only one oval.

0	1	2	3	4	5	6	7	8	9	10
◯	◯	◯	◯	◯	◯	◯	◯	◯	◯	◯

14. How would you rate the quality of your sleep?

(0 = very poor, 1 = poor, 2 = average, 3 = good, 4 = very good)

Mark only one oval.

	0	1	2	3	4	
Very	◯	◯	◯	◯	◯	Very good

Rate the severity of the following three symptoms over the past 24 hours.

(0 = none, 1-2 = mild, 3-4 = moderate, 5-6 = severe)

15. Headache

(0 = none, 1-2 = mild, 3-4 = moderate, 5-6 = severe)

Mark only one oval.

	0	1	2	3	4	5	6	
Non	◯	◯	◯	◯	◯	◯	◯	Severe

16. Nausea

(0 = none, 1-2 = mild, 3-4 = moderate, 5-6 = severe)

Mark only one oval.

	0	1	2	3	4	5	6	
Non	◯	◯	◯	◯	◯	◯	◯	Severe

17. Dizziness

(0 = none, 1-2 = mild, 3-4 = moderate, 5-6 = severe)

Mark only one oval.

	0	1	2	3	4	5	6	
Non	◯	◯	◯	◯	◯	◯	◯	Severe

18. In the past 24 hours, have you felt ill?

Mark only one oval.

- ◯ Yes - severe illness
- ◯ Yes - mild illness
- ◯ Yes - minor illness
- ◯ I have not felt ill

19. In the past 24 hours, have you vomited?

Mark only one oval.

- Yes
- No

20. In the past 24 hours, have you had a significant blow to the head ON THE FIELD?

Mark only one oval.

- Yes
- No

21. In the past 24 hours, have you had a significant blow to the head OFF OF THE FIELD?

Mark only one oval.

- Yes
- No

22. In the past 24 hours, have you been treated by your athletic trainer or a physician for any injuries?

Mark only one oval.

- Yes
- No

23. If have been treated for any injuries by your athletic trainer or a physician in the past 24 hours, please list the injury/location.

____

FOOD AND NUTRITION

REMINDER: All medical information provided will remain confidential
NOTE: 'Normal' indicates what has been typical for you personally over the past several weeks.

24. In the past 24 hours, approximately how much fiber have you consumed? (common foods containing large amounts of fiber: fruits, vegetables, whole grains, beans, nuts, seeds)

    (0 = almost none, 1 = less than normal, 2 = a normal amount, 3 = more than normal, 4 = far more than normal)

    Mark only one oval.

	0	1	2	3	4	
Alm	◯	◯	◯	◯	◯	Far more than normal

25. In the past 24 hours, approximately how much red meat have you consumed? (beef, pork, lamb, venison,)

    (0 = almost none, 1 = less than normal, 2 = a normal amount, 3 = more than normal, 4 = far more than normal)

    Mark only one oval.

	0	1	2	3	4	
Alm	◯	◯	◯	◯	◯	Far more than normal

26. In the past 24 hours, approximately how much refined carbohydrates and sugars have you consumed? (pasta, cereals, white bread, sweets)

(0 = almost none, 1 = less than normal, 2 = a normal amount, 3 = more than normal, 4 = far more than normal)

Mark only one oval.

	0	1	2	3	4	
Alm	◯	◯	◯	◯	◯	Far more than normal

27. In the past 24 hours, approximately how many diet sodas have you consumed?

Mark only one oval.

0	1	2	3	4
◯	◯	◯	◯	◯

28. In the past 24 hours, have you used NSAIDs (ibuprofen [Advil/Mortrin], naproxen [Aleve], etc.)?

NOTE: 'Normal' indicates what has been typical for you personally over the past several weeks.

Mark only one oval.

- ◯ Yes - a more than 'normal' amount
- ◯ Yes - a 'normal' dose
- ◯ Yes - a less than 'normal' amount
- ◯ None at all

29. In the past 24 hours, have you consumed products with caffeine (coffee, tea, energy drinks, pre-workout, etc.)?

NOTE: 'Normal' indicates what has been typical for you personally over the past several weeks.

Mark only one oval.

- Yes - a more than 'normal' amount
- Yes - a 'normal' amount
- Yes - a less than 'normal' amount
- None at all

30. In the past 24 hours, have you consumed any of the following nutritional supplements?

Check all that apply.

- [ ] Pro-biotics
- [ ] Fiber Supplements
- [ ] Protein Powder
- [ ] Creatine
- [ ] Pre-workout
- [ ] Multi-vitamin
- [ ] Fish Oil
- [ ] Vitamin D
- [ ] Collagen
- [ ] Other: ____________________

31. In the past 24 hrs, have you taken prescription drugs or medication?

Mark only one oval.

- Yes
- No

32. If you have taken any prescription drugs, please indicate the medication here.

REMINDER: All information provided will remain confidential.

33. In the past 24 hours, have you used any tobacco or nicotine products (cigarettes, chewing tobacco, zyn, vapes, etc.)?

NOTE: 'Normal' indicates what has been typical for you personally over the past several weeks.

Mark only one oval.

- Yes - a more than 'normal' amount
- Yes - a 'normal' amount
- Yes - a less than 'normal' amount
- None at all

34. In the past 24 hours, about how many alcoholic beverages have you consumed?

Select 10 drinks if you consumed more than 10 alcoholic beverages.

Mark only one oval.

0	1	2	3	4	5	6	7	8	9	10

35. In the past 24 hours, have you used cannabis products?

NOTE: 'Normal' indicates what has been typical for you personally over the past several weeks.

Mark only one oval.

- Yes - a more than 'normal' amount
- Yes - a 'normal' amount
- Yes - a less than 'normal' amount
- None at all

36. Please list any other recreation drugs used in the past 24 hours.

____

37. Other Notes

____

QUESTIONNAIRE SUBMISSION - PRESS SUBMIT

Thank you for completing your daily questionnaire. Please press submit to complete the questionnaire.

This content is neither created nor endorsed by Google.

Google Forms
