# Supplementary material for: Non-concussive head impacts sustained during American football correlate with changes in gut microbiome diversity and composition
Source: PLoS One. 2026 May 6;21(5):e0345651. doi: 10.1371/journal.pone.0345651 (PMC13148679; doi:10.1371/journal.pone.0345651)
Supplement: S3 File — A review of the concussion and microbiome response was conducted to identify taxa on which to focus in our examination of microbiome changes after non-concussive head impacts. The taxa used in our analysis of bacterial fluctuations after recorded impacts are highlighted in orange. (PDF) [file pone.0345651.s007.pdf]

Phylum	Class	Order	Family	Genus	Species	Model	Sample type	Experiment	Direction of change	Time until change	Time until recovery	Reference
Actinobacteria						humans	fecal	TBI patient with long-term impairments	increase	years	N/A	Urban et al., 2020
Actinomycetota	*Actinomycetes*	*Bifidobacteriales*	*Bifidobacteriaceae*	*Bifidobacterium*		Sprague Dawley rats	cecal	fluid percussion injury	decrease	4 days	N/A	Waligora-Dupriet et al., 2018
Actinomycetota	*Actinomycetes*	*Micrococcales*	*Microbacteriaceae*	*Anaerostipes*	*hadrus*	humans collegiate football players	fecal	in season vs. off season comparison	increase	months	N/A	Soriano et al., 2022
Actinomycetota	*Actinomycetes*	*Micrococcales*	*Micrococcaceae*	*Arthrobacter*	*sp. YN*	humans collegiate football players	fecal	in season vs. off season comparison	increase	months	N/A	Soriano et al., 2022
Actinomycetota	*Actinomycetes*	*Micrococcales*	*Micrococcaceae*	*Arthrobacter*		humans collegiate football players	fecal	in season vs. off season comparison	increase	months	N/A	Soriano et al., 2022
Actinomycetota	*Actinomycetes*	*Pseudonocardiales*	*Pseudonocardiaceae*			C57BL/6 mice	cecal	open-brain weight drop	increase	3 days	N/A	Ma et al., 2019
Actinomycetota	*Actinomycetes*					C57BL/6 mice	cecal	open-brain weight drop	increase	3 days	N/A	Ma et al., 2019
Bacillota (Firmicutes)	*Bacilli*	*Bacillales*	*Bacillaceae*	*Bacillus*	*cereus*	humans collegiate football players	fecal	concussed vs. non-concussed comparison	increase	months	N/A	Soriano et al., 2022
Bacillota (Firmicutes)	*Bacilli*	*Bacillales*	*Bacillaceae*	*Bacillus*	*thuringiensis*	humans collegiate football players	fecal	concussed vs. non-concussed comparison	increase	months	N/A	Soriano et al., 2022
Bacillota (Firmicutes)	*Bacilli*	*Bacillales*	*Bacillaceae*	*Bacillus*		humans collegiate football players	fecal	concussed vs. non-concussed comparison	increase	months	N/A	Soriano et al., 2022
Bacillota (Firmicutes)	*Bacilli*	*Caryophanales*				humans collegiate football players	fecal	in season vs. off season comparison	increase	months	N/A	Soriano et al., 2022
Bacillota (Firmicutes)	*Bacilli*	*Caryophanales*	*Alicyclobacillaceae*			humans collegiate football players	fecal	in season vs. off season comparison	increase	months	N/A	Soriano et al., 2022
Bacillota (Firmicutes)	*Bacilli*	*Caryophanales*	*Caryophanaceae*			C57BL/6 mice	cecal	open-brain weight drop	increase	3 days	N/A	Ma et al., 2019
Bacillota (Firmicutes)	*Bacilli*	*Caryophanales*	*Listeriaceae*	*Listeria*		humans collegiate football players	fecal	in season vs. off season comparison	increase	months	N/A	Soriano et al., 2022
Bacillota (Firmicutes)	*Bacilli*	*Caryophanales*	*Paenibacillaceae*	*Paenibacillus*	*mucilaginosus*	humans collegiate football players	fecal	in season vs. off season comparison	increase	months	N/A	Soriano et al., 2022
Bacillota (Firmicutes)	*Bacilli*	*Caryophanales*	*Paenibacillaceae*	*Paenibacillus*	*naphthalenovorans*	humans collegiate football players	fecal	in season vs. off season comparison	increase	months	N/A	Soriano et al., 2022
Bacillota (Firmicutes)	*Bacilli*	*Lactobacillales*	*Lactobacillaceae*	*Lactobacillus*	*gasser*	mice	fecal	open-brain controlled cortical impact	decrease	24 hrs	N/A	Treangen, 2018
Bacillota (Firmicutes)	*Bacilli*	*Lactobacillales*	*Lactobacillaceae*	*Lactobacillus*		Wistar rats	jejunum	multiple closed-brain weight drops	decrease	6 hrs, 2 days, or 30 days	N/A	Matharu, 2019
Bacillota (Firmicutes)	*Bacilli*	*Lactobacillales*	*Lactobacillaceae*	*Lactobacillus*		Sprague Dawley rats	cecal	fluid percussion injury	decrease	4 days	N/A	Waligora-Dupriet et al., 2018
Bacillota (Firmicutes)	*Bacilli*	*Lactobacillales*	*Leuconostocaceae*	*Leuconostoc*		humans collegiate football players	fecal	in season vs. off season comparison	increase	months	N/A	Soriano et al., 2022
Bacillota (Firmicutes)	*Bacilli*	*Lactobacillales*	*Leuconostocaceae*	*Leuconostoc*		Sprague Dawley rats	cecal	fluid percussion injury	decrease	4 days	N/A	Waligora-Dupriet et al., 2018
Bacillota (Firmicutes)	*Bacilli*	*Lactobacillales*	*Leuconostocaceae*	*Weissella*		humans collegiate football players	fecal	in season vs. off season comparison	increase	months	N/A	Soriano et al., 2022
Bacillota (Firmicutes)	*Bacilli*	*Lactobacillales*	*Streptococcaceae*	*Lactococcus*	*Lactococcus piscium*	humans collegiate football players	fecal	in season vs. off season comparison	increase	months	N/A	Soriano et al., 2022
Bacillota (Firmicutes)	*Bacilli*	*Lactobacillales*	*Streptococcaceae*	*Lactococcus*		humans collegiate football players	fecal	in season vs. off season comparison	increase	months	N/A	Soriano et al., 2022
Bacillota (Firmicutes)	*Bacilli*	*Lactobacillales*	*Streptococcaceae*	*Streptococcus*		Wistar rats	jejunum	multiple closed-brain weight drops	decrease	6 hrs, 2 days, or 30 days	N/A	Matharu, 2019
Bacillota (Firmicutes)	*Bacilli*	*Lactobacillales*	*Streptococcaceae*			C57BL/6 mice	cecal	open-brain weight drop	increase	3 days	N/A	Ma et al., 2019
Bacillota (Firmicutes)	*Clostridia*	*Clostridiales*	*Oscillospiraceae (Ruminococcus)*	*Ruminococcus*	*flavefaciens*	mice	fecal	open-brain controlled cortical impact	decrease	24 hrs	N/A	Treangen, 2018
Bacillota (Firmicutes)	*Clostridia*	*Clostridiales*	*Oscillospiraceae (Ruminococcus)*			Sprague Dawley rats	fecal	open-brain controlled cortical impact	decrease	24 hrs	N/A	Nicholson et al., 2019
Bacillota (Firmicutes)	*Clostridia*	*Clostridiales*	*Oscillospiraceae (Ruminococcus)*			humans	fecal	TBI patient with long-term impairments	increase	years	N/A	Urban et al., 2020
Bacillota (Firmicutes)	*Clostridia*	*Clostridiales*	*Oscillospiraceae (Ruminococcus)*			C57BL6/J mice	fecal	open-brain controlled cortical impact	decrease	24hr, 3d, 7d, 14d, 28d	N/A	Opeyemi et al., 2023
Bacillota (Firmicutes)	*Clostridia*	*Eubacteriales*	*Oscillospiraceae (Ruminococcus)*	*Ethanoligenes*	*harbinense*	humans collegiate football players	fecal	concussed vs. non-concussed comparison	increase	months	N/A	Soriano et al., 2022
Bacillota (Firmicutes)	*Clostridia*	*Eubacteriales*	*Oscillospiraceae (Ruminococcus)*	*Flavonifractor*	*plautii*	humans collegiate football players	fecal	concussed vs. non-concussed comparison	increase	months	N/A	Soriano et al., 2022
Bacillota (Firmicutes)	*Clostridia*	*Eubacteriales*	*Oscillospiraceae (Ruminococcus)*	*Mageeibacillus*	*indolicus*	humans collegiate football players	fecal	concussed vs. non-concussed comparison	increase	months	N/A	Soriano et al., 2022
Bacillota (Firmicutes)	*Clostridia*	*Eubacteriales*	*Oscillospiraceae (Ruminococcus)*	*unclassified Oscillospiraceae*	*Ruminococcaceae bacterium CPB6*	humans collegiate football players	fecal	concussed vs. non-concussed comparison	increase	months	N/A	Soriano et al., 2022
Bacillota (Firmicutes)	*Clostridia*	*Eubacteriales*	*Peptococcaceae*	*Desulfotomaculum*		humans collegiate football players	fecal	in season vs. off season comparison	increase	months	N/A	Soriano et al., 2022
Bacillota (Firmicutes)	*Clostridia*	*Clostridiales*	*Clostridiaceae*	*Clostridium*		mice	fecal	open-brain controlled cortical impact	increase	24 hrs	N/A	Treangen, 2018
Bacillota (Firmicutes)	*Clostridia*	*Clostridiales*	*Clostridiaceae*	*Clostridium*		Sprague Dawley rats	cecal	fluid percussion injury	decrease	4 days	N/A	Waligora-Dupriet et al., 2018
Bacillota (Firmicutes)	*Clostridia*	*Eubacteriales*	*Eubacteriaceae*	*Eubacterium*	*rectale*	humans collegiate football players	fecal	concussed vs. non-concussed comparison	decrease	months	N/A	Soriano et al., 2022
Bacillota (Firmicutes)	*Clostridia*	*Eubacteriales*	*Eubacteriaceae*	*Eubacterium*	*sulci*	mice	fecal	open-brain controlled cortical impact	increase	24 hrs	N/A	Treangen, 2018
Bacillota (Firmicutes)	*Clostridia*	*Eubacteriales*	*Eubacteriaceae*	*Eubacterium*	*ventriosum*	mice	fecal	open-brain controlled cortical impact	decrease	24 hrs	N/A	Treangen, 2018
Bacillota (Firmicutes)	*Clostridia*	*Lachnospirales*	*Lachnospiraceae*	*Anaerostipes*	*hadrus*	humans collegiate football players	fecal	concussed vs. non-concussed comparison	decrease	months	N/A	Soriano et al., 2022
Bacillota (Firmicutes)	*Clostridia*	*Lachnospirales*	*Lachnospiraceae*	*Marvinbryantia*	*formatexigens*	mice	fecal	open-brain controlled cortical impact	increase	24 hrs	N/A	Treangen, 2018
Bacillota (Firmicutes)	*Clostridia*	*Lachnospirales*	*Lachnospiraceae*	*Marvinbryantia*		mice	fecal	open-brain controlled cortical impact	increase	24 hrs	N/A	Treangen, 2018
Bacillota (Firmicutes)	*Clostridia*	*Lachnospirales*	*Lachnospiraceae*			humans collegiate football players	fecal	concussed vs. non-concussed comparison	decrease	months	N/A	Soriano et al., 2022
Bacillota (Firmicutes)	*Clostridia*	*Lachnospirales*	*Lachnospiraceae*			Sprague Dawley rats	fecal	open-brain controlled cortical impact	decrease	2 hrs	7 days	Nicholson et al., 2019
Bacillota (Firmicutes)	*Clostridia*	*Lachnospirales*	*Lachnospiraceae*			C57BL6/J mice	fecal	open-brain controlled cortical impact	decrease	24hr, 3d, 7d, 14d, 28d	N/A	Opeyemi et al., 2022
Bacillota (Firmicutes)	*Clostridia*	*Eubacteriales*	*Mogibacteriaceae*			Sprague Dawley rats	fecal	open-brain controlled cortical impact	decrease	24 hrs	7 days	Nicholson et al., 2019
Bacillota (Firmicutes)	*Clostridia*	*Eubacteriales*	*Oscillospiraceae*			humans collegiate football players	fecal	concussed vs. non-concussed comparison	increase	months	N/A	Soriano et al., 2022
Bacillota (Firmicutes)	*Clostridia*	*Halanaerobiales*	*Halanaerobiaceae*	*Halothermothrix*		humans collegiate football players	fecal	in season vs. off season comparison	increase	months	N/A	Soriano et al., 2022
Bacillota (Firmicutes)	*Clostridia*	*Halanaerobiales*	*Halobacteroidaceae*			humans collegiate football players	fecal	in season vs. off season comparison	increase	months	N/A	Soriano et al., 2022
Bacillota (Firmicutes)	*Clostridia*	*Thermoanaerobacterales*	*Thermoanaerobacteraceae*	*Thermoanaerobacterium*		humans collegiate football players	fecal	in season vs. off season comparison	increase	months	N/A	Soriano et al., 2022
Bacillota (Firmicutes)	*Clostridia*	*Thermoanaerobacterales*	*Thermoanaerobacteraceae*			humans collegiate football players	fecal	in season vs. off season comparison	increase	months	N/A	Soriano et al., 2022
Bacillota (Firmicutes)	*Erysipelotrichia*	*Erysipelotrichales*	*Erysipelotrichaceae*			C57BL6/J mice	fecal	open-brain controlled cortical impact	increase	24hr, 3d, 7d, 14d, 28d	N/A	Opeyemi et al., 2025
Bacillota (Firmicutes)	*Tissierellia*	*Tissierellales*	*Peptoniphilaceae*	*Anaerococcus*	*prevotii*	humans collegiate football players	fecal	concussed vs. non-concussed comparison	increase	months	N/A	Soriano et al., 2022

Bacillota (Firmicutes)						humans	fecal	TBI patient with long-term impairments	increase	years	N/A	Urban et al., 2020
Bacteroidota	*Bacteroidia*	*Bacteroidales*	*Bacteroidaceae*	*Bacteroides*		Sprague Dawley rats	cecal	fluid percussion injury	decrease	4 days	N/A	Waligora-Dupriet et al., 2018
Bacteroidota	*Bacteroidia*	*Bacteroidales*	*Bacteroidaceae*	*Bacteroides*		humans collegiate football players	fecal	concussed vs. non-concussed comparison	increase	months	N/A	Soriano et al., 2022
Bacteroidota	*Bacteroidia*	*Bacteroidales*	*Bacteroidaceae*			C57BL/6 mice	cecal	open-brain weight drop	increase	3 days	N/A	Ma et al., 2019
Bacteroidota	*Bacteroidia*	*Bacteroidales*	*Bacteroidaceae*			Sprague Dawley rats	fecal	open-brain controlled cortical impact	increase	24 hrs	7 days	Nicholson et al., 2019
Bacteroidota	*Bacteroidia*	*Bacteroidales*	*Bacteroidaceae*			C57BL6/J mice	fecal	open-brain controlled cortical impact	decrease	24hr, 3d, 7d, 14d, 28d	N/A	Opeyemi et al., 2021
Bacteroidota	*Bacteroidia*	*Bacteroidales*	*Odoribacteraceae*			humans collegiate football players	fecal	in season vs. off season comparison	increase	months	N/A	Soriano et al., 2022
Bacteroidota	*Bacteroidia*	*Bacteroidales*	*Porphyromonadaceae*			C57BL/6 mice	cecal	open-brain weight drop	increase	3 days	N/A	Ma et al., 2019
Bacteroidota	*Bacteroidia*	*Bacteroidales*	*Prevotellaceae*			Sprague Dawley rats	cecal	fluid percussion injury	decrease	4 days	N/A	Waligora-Dupriet et al., 2018
Bacteroidota	*Bacteroidia*	*Bacteroidales*	*Prevotellaceae*	*Prevotella*		humans collegiate football players	fecal	in season vs. off season comparison	increase	months	N/A	Soriano et al., 2022
Bacteroidota	*Bacteroidia*	*Bacteroidales*	*Prevotellaceae*			humans	fecal	TBI patient with long-term impairments	decrease	years	N/A	Urban et al., 2020
Bacteroidota	*Bacteroidia*	*Bacteroidales*	*Rikenellaceae*			C57BL/6 mice	cecal	open-brain weight drop	increase	3 days	N/A	Ma et al., 2019
Bacteroidota						humans	fecal	TBI patient with long-term impairments	decrease	years	N/A	Urban et al., 2020
Campylobacter	*Epsilonproteobacteria*	*Campylobacterales*	*Campylobacteraceae*	*Campylobacter*		Wistar rats	jejunum	multiple closed-brain weight drops	increase	6 hrs, 2 days, or 30 days	N/A	Matharu, 2019
Campylobacter	*Epsilonproteobacteria*	*Campylobacterales*	*Helicobacteraceae*			C57BL/6 mice	cecal	open-brain weight drop	decrease	3 days	N/A	Ma et al., 2019
Chrysiogenota	*Chrysiogenetes*	*Chrysiogenales*	*Chrysiogenaceae*	*Desulfurispirillum*	*indicum*	humans collegiate football players	fecal	in season vs. off season comparison	increase	months	N/A	Soriano et al., 2022
Chrysiogenota	*Chrysiogenetes*	*Chrysiogenales*	*Chrysiogenaceae*	*Desulfurispirillum*		humans collegiate football players	fecal	in season vs. off season comparison	increase	months	N/A	Soriano et al., 2022
Cyanobacteria	*4C0d-2*					C57BL/6 mice	cecal	open-brain weight drop	decrease	3 days	N/A	Ma et al., 2019
Deferribacteres	*Deferribacteres*	*Deferribacterales*	*Deferribacteraceae*			Sprague Dawley rats	fecal	open-brain controlled cortical impact	decrease	24 hrs	7 days	Nicholson et al., 2019
Deinococcota	*Deinococci*	*Deinococcales*	*Deinococcaceae*			humans collegiate football players	fecal	in season vs. off season comparison	increase	months	N/A	Soriano et al., 2022
Deinococcota	*Deinococci*	*Trueperales*	*Trueperaceae*	*Truepera*		humans collegiate football players	fecal	in season vs. off season comparison	increase	months	N/A	Soriano et al., 2022
Mycoplasmatota	*Mollicutes*	*Anaeroplasmatales*	*Anaeroplasmataceae*			Sprague Dawley rats	fecal	open-brain controlled cortical impact	decrease	2 hrs	N/A	Nicholson et al., 2019
Mycoplasmatota	*Mollicutes*	*Entomoplasmatales*	*Spiroplasmataceae*			humans collegiate football players	fecal	in season vs. off season comparison	increase	months	N/A	Soriano et al., 2022
Mycoplasmatota	*Mollicutes*	*Mycoplasmatales*	*Mycoplasmataceae*	*Mycoplasma*	*mobile*	humans collegiate football players	fecal	in season vs. off season comparison	increase	months	N/A	Soriano et al., 2022
Mycoplasmatota	*Mollicutes*	*Mycoplasmatales*	*Mycoplasmataceae*	*Mycoplasma*		humans collegiate football players	fecal	in season vs. off season comparison	increase	months	N/A	Soriano et al., 2022
Pseudomonadota	*Alphaproteobacteria*	*Hyphomicrobiales*	*Brucellaceae*			C57BL/6 mice	cecal	open-brain weight drop	increase	3 days	N/A	Ma et al., 2019
Pseudomonadota	*Alphaproteobacteria*	*Hyphomicrobiales*	*Methylobacteriaceae,*			C57BL/6 mice	cecal	open-brain weight drop	increase	3 days	N/A	Ma et al., 2019
Pseudomonadota	*Alphaproteobacteria*					C57BL/6 mice	cecal	open-brain weight drop	increase	3 days	N/A	Ma et al., 2019
Pseudomonadota	*Betaproteobacteria*	*Burkholderiales*	*Burkholderiaceae*	*Burkholderia*		humans collegiate football players	fecal	in season vs. off season comparison	increase	months	N/A	Soriano et al., 2022
Pseudomonadota	*Betaproteobacteria*	*Burkholderiales*	*Burkholderiaceae*			humans collegiate football players	fecal	in season vs. off season comparison	increase	months	N/A	Soriano et al., 2022
Pseudomonadota	*Betaproteobacteria*	*Burkholderiales*	*Comamonadaceae*			C57BL/6 mice	cecal	open-brain weight drop	increase	3 days	N/A	Ma et al., 2019
Pseudomonadota	*Betaproteobacteria*	*Burkholderiales*	*unclassified Burkholderiales*			C57BL/6 mice	cecal	open-brain weight drop	increase	3 days	N/A	Ma et al., 2019
Pseudomonadota	*Betaproteobacteria*	*Neisseriales*	*Neisseriaceae*			humans collegiate football players	fecal	in season vs. off season comparison	increase	months	N/A	Soriano et al., 2022
Campylobacter	*Epsilonproteobacteria*	*Campylobacterales*	*Helicobacteraceae*			Wistar rats	jejunum	multiple closed-brain weight drops	increase	6 hrs, 2 days, or 30 days	N/A	Matharu, 2019
Pseudomonadota	*Epsilonproteobacteria*					C57BL/6 mice	cecal	open-brain weight drop	decrease	3 days	N/A	Ma et al., 2019
Pseudomonadota	*Gammaproteobacteria*	*Enterobacterales*	*Enterobacteriaceae*	*Escherichia*	*coli*	Sprague Dawley rats	cecal	fluid percussion injury	increase	4 days	N/A	Waligora-Dupriet et al., 2018
Pseudomonadota	*Gammaproteobacteria*	*Enterobacterales*	*Enterobacteriaceae*			humans	cultured rectal swab	severe TBI hospitalized	increase	0, 3, and 7 days	N/A	Mahajan, 2023
Pseudomonadota	*Gammaproteobacteria*	*Enterobacterales*	*Enterobacteriaceae*			Sprague Dawley rats	fecal	open-brain controlled cortical impact	increase	3 days	7 days	Nicholson et al., 2019
Pseudomonadota	*Gammaproteobacteria*	*Enterobacterales*	*Hafniaceae*	*Edwardsiella*	*ictaluri*	humans collegiate football players	fecal	in season vs. off season comparison	increase	months	N/A	Soriano et al., 2022
Pseudomonadota	*Gammaproteobacteria*	*Enterobacterales*	*Hafniaceae*	*Edwardsiella*		humans collegiate football players	fecal	in season vs. off season comparison	increase	months	N/A	Soriano et al., 2022
Pseudomonadota	*Gammaproteobacteria*	*Enterobacterales*	*Yersiniaceae*			humans collegiate football players	fecal	in season vs. off season comparison	increase	months	N/A	Soriano et al., 2022
Pseudomonadota	*Gammaproteobacteria*	*Immundisolibacterales*	*Solibacteraceae*			humans collegiate football players	fecal	in season vs. off season comparison	increase	months	N/A	Soriano et al., 2022
Pseudomonadota	*Gammaproteobacteria*	*Pseudomonadales*	*Moraxellaceae*			C57BL/6 mice	cecal	open-brain weight drop	increase	3 days	N/A	Ma et al., 2019
Pseudomonadota	*Gammaproteobacteria*	*Pseudomonadales*	*Moraxellaceae*			humans collegiate football players	fecal	in season vs. off season comparison	increase	months	N/A	Soriano et al., 2022
Pseudomonadota	*Gammaproteobacteria*	*Pseudomonadales*	*Pseudomonadaceae*	*Pseudomonas*		humans collegiate football players	fecal	in season vs. off season comparison	increase	months	N/A	Soriano et al., 2022
Pseudomonadota	*Gammaproteobacteria*	*Pseudomonadales*	*Pseudomonadaceae*			Sprague Dawley rats	fecal	open-brain controlled cortical impact	increase	3 days	7 days	Nicholson et al., 2019
Pseudomonadota	*Gammaproteobacteria*	*Pseudomonadales*	*Pseudomonadaceae*			C57BL/6 mice	cecal	open-brain weight drop	increase	3 days	N/A	Ma et al., 2019
Pseudomonadota	*Gammaproteobacteria*					C57BL/6 mice	cecal	open-brain weight drop	increase	3 days	N/A	Ma et al., 2019
Pseudomonadota	*Oligoflexia*	*Bacteriovoracales*	*Halobacteriovoraceae*	*Halobacteriovorax*	*marinus*	humans collegiate football players	fecal	in season vs. off season comparison	increase	months	N/A	Soriano et al., 2022
Verrucomicrobia	*Verrucomicrobiae*	*Verrucomicrobiales*	*Listeriaceae*			Sprague Dawley rats	fecal	open-brain controlled cortical impact	decrease	24 hrs	7 days	Nicholson et al., 2019
Verrucomicrobia	*Verrucomicrobiae*	*Verrucomicrobiales*	*Verrucomicrobiaceae*			C57BL6/J mice	fecal	open-brain controlled cortical impact	increase	24hr, 3d, 7d, 14d, 28d	N/A	Opeyemi et al., 2024
Verrucomicrobia						humans	fecal	TBI patient with long-term impairments	increase	years	N/A	Urban et al., 2020
